# Plants’ molecular behavior to heavy metals: from criticality to toxicity

**DOI:** 10.3389/fpls.2024.1423625

**Published:** 2024-08-30

**Authors:** Ahmed H. El-Sappah, Yumin Zhu, Qiulan Huang, Bo Chen, Salma A. Soaud, Mohamed A. Abd Elhamid, Kuan Yan, Jia Li, Khaled A. El-Tarabily

**Affiliations:** ^1^ College of Agriculture, Forestry, and Food Engineering, Yibin University, Yibin, Sichuan, China; ^2^ Department of Genetics, Faculty of Agriculture, Zagazig University, Zagazig, Egypt; ^3^ Department of Biology, College of Science, United Arab Emirates University, Al Ain, United Arab Emirates

**Keywords:** agricultural productivity, cross-tolerance, genotoxicity, hormesis, molecular responses, transport genes

## Abstract

The contamination of soil and water with high levels of heavy metals (HMs) has emerged as a significant obstacle to agricultural productivity and overall crop quality. Certain HMs, although serving as essential micronutrients, are required in smaller quantities for plant growth. However, when present in higher concentrations, they become very toxic. Several studies have shown that to balance out the harmful effects of HMs, complex systems are needed at the molecular, physiological, biochemical, cellular, tissue, and whole plant levels. This could lead to more crops being grown. Our review focused on HMs’ resources, occurrences, and agricultural implications. This review will also look at how plants react to HMs and how they affect seed performance as well as the benefits that HMs provide for plants. Furthermore, the review examines HMs’ transport genes in plants and their molecular, biochemical, and metabolic responses to HMs. We have also examined the obstacles and potential for HMs in plants and their management strategies.

## 1 Introduction

Plants, similar to other living species, are vulnerable to high levels of heavy metals (HMs) in the atmosphere, resulting from both human activities and environmental factors (El-Sappah and Rather, 2022; Li et al., 2023). The poisoning of the environment with HMs is mostly caused by intensive mining operations, fast industrialization, and widespread agricultural activities (Adnan et al., 2024). The presence of high levels of HMs in soil and water is a notable illustration of human activities that have a substantial impact on the environment and constitute a considerable hazard (El-Sappah et al., 2021a; Hama Aziz et al., 2023). HMs may be transferred over long distances in both gaseous and particle forms, leading to their rapid buildup in biological systems, water, and sediment (Mishra et al., 2017).

A total of 53 elements have been classified as HMs based on their density, which exceeds 5 g/cm^3^ (Ali and Khan, 2018). For the essential metabolic operations of plant cells, a total of 17 elements are required. However, only six of these elements are classified as HMs: copper (Cu), zinc (Zn), manganese (Mn), iron (Fe), molybdenum (Mo), and nickel (Ni). The macroelements are carbon (C), oxygen (O), hydrogen (H), magnesium (Mg), sulfur (S), nitrogen (N), calcium (Ca), phosphorus (P), and potassium (K), while the microelements are Cu, Zn, Mn, Mo, boron (B), and chlorine (Cl) (Fan et al., 2021). In plants, the macro- and microelements are essential for the regulation of numerous physiological and biochemical processes, such as chlorophyll formation, photosynthesis, nucleic acid metabolism, protein modification, intra-compartmental redox reactions, carbohydrate metabolism, and N fixation (Dutta et al., 2018; Zayed et al., 2023).

It is intriguing that while numerous HMs function as microelements, others, such as aluminum (Al), cadmium (Cd), chromium (Cr), lead (Pb), and mercury (Hg), have detrimental effects on plants. These consequences include impaired photosynthesis, chlorosis, decreased biomass output, disrupted water balance, and impaired nutrient absorption (Angulo-Bejarano et al., 2021). The unpreceded use of agrochemicals, long-term application of municipal sewage effluent, industrial waste disposal, waste incineration, and vehicle exhausts are the primary sources of HMs in agricultural soils (Mishra et al., 2017).

Plants ingest and accumulate HMs in soil with high concentrations, which subsequently reach human nutrition through the food chain (Angon et al., 2024). The absorption of HMs by both underground and above-ground surfaces of plants can have a direct or indirect impact on plant health (Emamverdian et al., 2015). The inhibition of cytoplasmic enzymes and the injury to cell structures are the direct consequences of oxidative stress (Jadia and Fulekar, 2009). An often observed result of HM toxicity is the overabundance of reactive oxygen species (ROS) and methylglyoxal (MG), both of which can lead to lipid peroxidation, protein oxidation, enzyme deactivation, DNA damage, disruption of ionic balance in plant cells, and/or interaction with other essential components of plant cells (Hossain et al., 2012; Jomova et al., 2023).

Some HMs indirectly impose oxidative stress through a variety of mechanisms, including the depletion of glutathione, the binding of sulfhydryl groups of proteins (Jaishankar et al., 2014), the inhibition of antioxidative enzymes, or the induction of ROS-producing enzymes such as NADPH oxidases (Bielen et al., 2013). Regardless of whether it is direct or indirect, plants that are exposed to high levels of HMs experience a reduction or even the complete cessation of all metabolic activities (Singh et al., 2015).

Plant cells react to the toxicity caused by HMs via complex and interrelated systems that operate at many levels and include both immediate and long-lasting processes (Dutta et al., 2018). The immediate or short-term reactions include the rapid modification of the rates at which hundreds or even thousands of genes are transcribed, followed by alterations in physiological and metabolic processes (Dutta et al., 2018). On the other hand, genetic alterations and epigenetic modifications are associated with enduring reactions (Ryu et al., 2015). The control of gene expression, which is an essential part of the plant’s response to stress, usually involves making changes to the levels of stress-responsive genes in a way that is both common to all plants and specific to each individual plant (Gallo-Franco et al., 2020).

Therefore, it is logical to expect that plants will react to HMs’ toxicity, which causes both oxidative and genotoxic effects, by organizing and combining different elements of stress perception and signaling networks, with the possibility of communication at different stages, depending on the circumstances (Dutta et al., 2018). The environmental, ecological, and genetic effects of HMs on plants, as well as their resources, occurrence, and agroecological ramifications, were discussed in the current review. The challenges and prospects of HMs’ impacts on plants, as well as the methods for mitigating them, have also been the subject of discussion.

## 2 HMs in plants: resources, occurrence, and agroecological ramifications

The presence of HMs in soil may have negative effects on human and animal health as well as on soil quality, fertility, and agricultural productivity (Rashid et al., 2023). Given the fast-paced changes in the economy and culture, many hazardous materials found in polluted soil constitute a risk to both the general people and the environment (Hajam et al., 2023). Cd, Hg, Cu, Zn, Ni, Pb, Cr, and arsenic (As) are often detected as contaminants in soil settings (Priya et al., 2023). This kind of pollution poses a significant biological risk, is widely spread, and is prevalent in the soil environment (Adnan et al., 2024). The concentration of hazardous materials in the soil is beyond the acceptable threshold in five million areas worldwide (Rodríguez Eugenio et al., 2018).

According to the Environmental Protection Agency (EPA) (Goyer et al., 2004), Hg, Pb, Cd, and As are the most dangerous metals/metalloids in the environment. Human activities, such as the use of fertilizers in agriculture, the manufacturing of compounds, and the extraction of minerals, are the main causes of the creation of hazardous substances in soil (Tang et al., 2019). Multiple studies have shown that natural sources of HMs in the environment are often of lesser importance when compared to human activities (Dixit et al., 2015). There are two main origins of HMs: natural and anthropogenic (Angon et al., 2024). The HMs are mostly derived from volcanic and sedimentary minerals, making them the most abundant natural sources (Alengebawy et al., 2021).

The main origin of HMs in soils is the parent material from which they were first generated (Angon et al., 2024). Sedimentary rocks make up around 5% of the Earth’s mantle, whereas igneous elements make up 95% (Sarwar et al., 2017). On the other hand, the phrase “anthropogenic” usually refers to sources that are created by humans. Anthropogenic activities, such as burning fossil fuels for electricity, disposing of municipal waste, applying fertilizer, using pesticides, and irrigating with effluent, increase the levels of HMs in agricultural soil settings (Angon et al., 2024).

Effective soil management is a crucial aspect of sustainable agriculture, with soil biology playing a vital role in this context (Srivastava et al., 2017). Soil microorganisms are essential components of the ecosystem (Jiang and Li, 2020). Microorganisms play a crucial role in maintaining soil fertility by breaking down organic matter and cycling nutrients (Wu et al., 2024). However, stressors such as excessive temperature, pH, salinity, and chemical pollution might have a negative impact on them (Paz-Ferreiro and Fu, 2016). As the quantity of HMs grows, the capacity of microorganisms to survive declines (Igiri et al., 2018).

**Figure 1:**
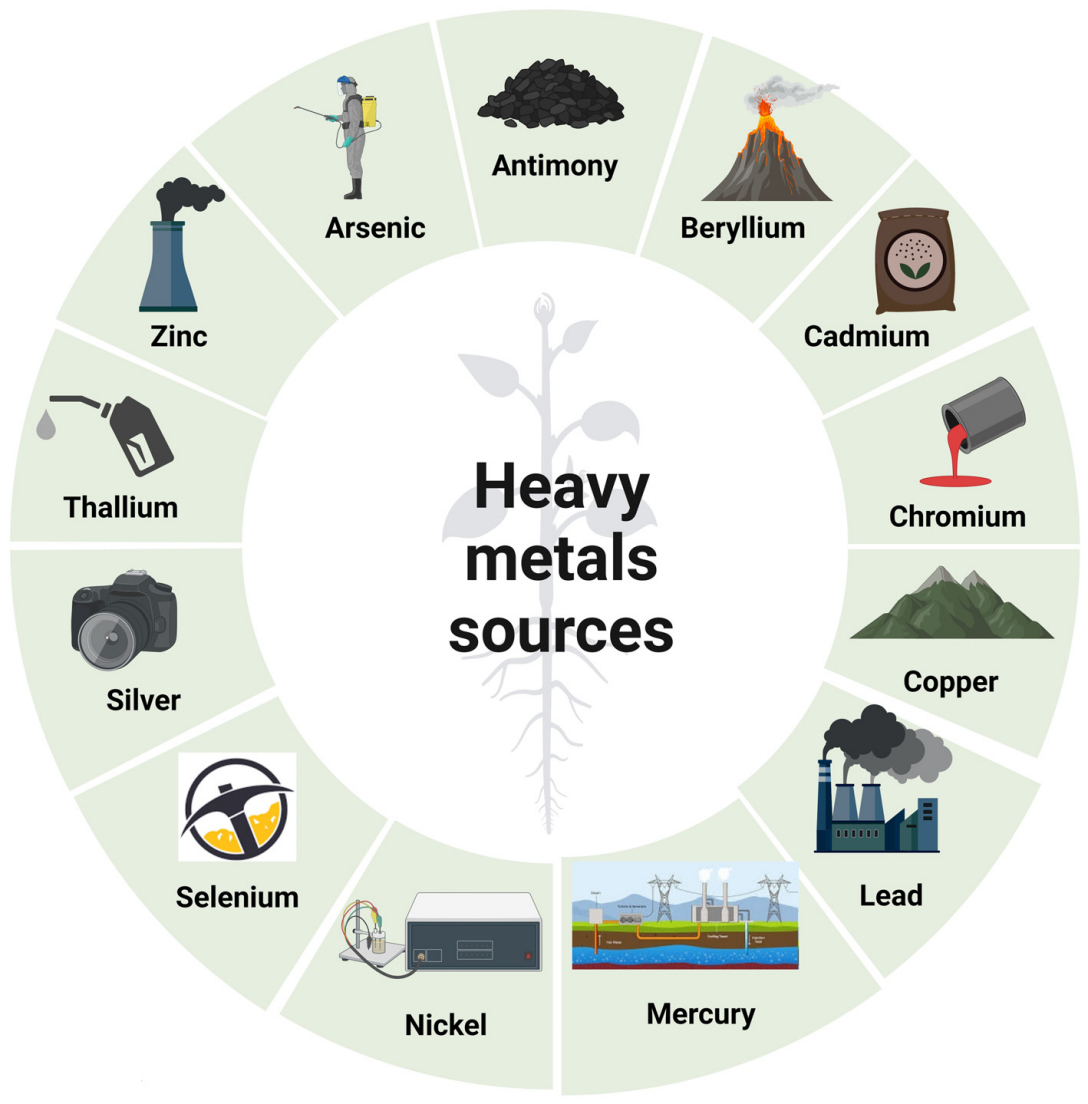
Various origins of heavy metals. Coal combustion, mining, refining, soil erosion, and volcanic eruptions are all sources of antimony. Sources of arsenic include smelting, mining, atmospheric deposition, pesticides, and geological sedimentation. Volcanic dust and coal and hydrocarbon combustion are sources of beryllium as well. Sources of cadmium include plastic, fertilizer, pesticides, refining, and welding. Sources of chromium include textiles, dyeing, electroplating, paint manufacturing, steel fabrication, and tanning. Copper is obtained through mining, refining, painting, plating, and printing. Coal combustion, electroplating, battery manufacturing, mining, paint, and pigments are all lead sources. Batteries, coal combustion, geothermal activities, mining, paint and paper industries, volcanic eruptions, and geological weathering are all sources of mercury. Sources of nickel include porcelain enameling, electroplating, non-ferrous metals, and pigments. Sources of selenium include coal combustion and mining. Sources of silver include the production of batteries, mining, photographic processing, and smelting. Production of cement, combustion of fossil fuels, metal smelting, and hydrocarbon refining are all sources of thallium. Brass manufacturing, mining, hydrocarbon refining, and plumbing are all sources of zinc. This figure was made using BioRender.

The addition of Pb–Cu slurry, Pb–Cu dust, Pb–Zn dust, and Cd–Pb–Zn to forest soil resulted in a reduction in the number of colony-forming units (CFUs) of bacteria and fungi (Srivastava et al., 2017). Generally, the impact of low levels of HMs on soil respiration is minimal (Verma et al., 2010). However, when HMs’ pollution or toxicity intensifies, this effect becomes less significant. The introduction of HMs may either enhance or hinder N-mineralization, which can be related to differences in the experimental approach, variances in soil parameters, and substrate concentrations (Dai et al., 2004). HMs’ pollution generally has a negative impact on N transformation processes, which, in turn, affects N-mineralization (Dai et al., 2004; Hamsa et al., 2017). The bioavailability of metals in soils is influenced by factors such as metal concentrations, soil pH, organic matter, and sediment content (Rieuwerts et al., 1998). HMs play a crucial role in controlling the activities of various soil enzymes, such as arylsulfatase, alkaline phosphatase, b-glucosidase, cellulase, dehydrogenase, invertase, protease, and urease (Aponte et al., 2020). **Figure 1** depicts the many sources of HMs (Angon et al., 2024).

## 3 Plant response to HM exposure

The persistence of toxic HMs in the soil ecosystem is a considerable hazard for living animals and plants (Kraemer, 2009; Abd Elnabi et al., 2023). The plant roots serve as the main points of contact for terrestrial plants to be exposed to harmful HMs (Podar and Maathuis, 2022). Plants have developed new, adaptive, and precise methods to withstand the harmful effects of HM stress (Tiwari and Lata, 2018). This mechanism involves various strategies such as immobilization, exclusion outside the plasma membrane, restriction of absorption and transport, synthesis of specific HM transporters, induction of stress proteins, and chelation and sequestration by specific ligands (Clemens, 2001; DalCorso et al., 2008; Adrees et al., 2015).

To maintain a low concentration of metal ions in the cytoplasm, it is feasible to block the transport of hazardous metals across the plasma membrane, which is the cellular mechanism for HMs’ tolerance (Hall, 2002). Here are two direct approaches. The objective may be achieved by either augmenting the attachment of metal ions to the cell wall or expelling the metal from the cell using active efflux pumps. Another approach to detoxification includes the process of chelation or modifying the concentration of harmful metal ions to a lower level, thereby rendering them inactive (Tong et al., 2004). Several factors, such as plant structure, plant life cycle, plant vigor, soil pH, root system depth, temperature, partial oxygen pressure, carbohydrate level, respiration rate, nutrient interface, and microbial presence, have a significant impact on the accumulation of metals in plants (Chen et al., 2006).

Plants have the ability to cause HMs to form negatively charged particles by changing the pH of the soil around their roots or by releasing negatively charged ions such as PO_4_^3−^. During the process of adsorption, the surface of the root has the ability to bind a substantial amount of HMs. The accumulation of these HMs [Cd, Ni, strontium (sr), and Pb] in plant root tissues happens quickly (Hossain et al., 2012). The plants have been categorized into three categories based on their survival strategies under adverse conditions: accumulators, excluders, and indicators (Baker, 1981). Plants undergo hyperaccumulation of HMs, resulting in the accumulation of metals exceeding 0.1%–1% of the dry weight. The term “hyperaccumulator” was used by Baker and Brooks (1989) to refer to plants that have a leaf nickel concentration above 1,000 mg/g. Hyperaccumulator species refer to plants that have the ability to collect more than 100 mg of Cd per kilogram or more than 500 mg of Cr per kilogram in dried plant leaf tissue (Kumar et al., 2019). A plant with a hyperaccumulator trait is capable of accumulating and enduring significant levels of metal pollution.

Some plant species have the ability to flourish in soil that is polluted with HMs and may collect substantial levels of metals in such soil (Lombi et al., 2002). The primary methods involved in the hyperaccumulation of toxic metals in plants include bio-activation of HMs in the rhizosphere through root microbe interfaces, enhanced activity of metal conveyor proteins in cell membranes, detoxification of metals by restricting them to apoplasts, chelation of HMs in the cytoplasm by multiple ligands, and sequestration of metals into the vacuole by multiple ligands (Kumar et al., 2019).

## 4 The performance of seeds and seedlings under HM stress

While the seed coat initially offers some defense against metal stress before germination, it will gradually rupture or become more porous throughout the germination process (Kranner and Colville, 2011). Current data suggests that metals have two distinct impacts on seed germination: their overall toxicity and their ability to hinder the uptake of water (Osman and Fadhlallah, 2023). Pb significantly affects the physical and biological characteristics of seeds, hindering their ability to sprout, the roots to grow, the seedlings to develop, the plants to grow, water to be transported, chlorophyll to be produced, and protein to be synthesized (Collin et al., 2022).

Pb also hampers the production of ATP, causes the oxidation of lipids, and leads to DNA damage, culminating in an accumulation of ROS (Pourrut et al., 2011; Ur Rahman et al., 2024). Soils contaminated with Pb hinder the growth of seedlings by causing an increase in lipid peroxidation and the activation of enzymes such as superoxide dismutase, guaiacol peroxidase, ascorbate peroxidase, and glutathione (GSH)–ascorbate cycle enzymes (Sethy and Ghosh, 2013).

Cd is known for its capacity to inhibit seed germination via several methods. It has a negative impact on metabolic reactivation by decreasing the number of hydrolyzing enzymes, hindering starch mobilization, and preventing seed imbibition. It may also influence signaling via Ca, mitogen-activated protein kinases (MAPKs), and transcription factors (TFs) as well as the levels of phytohormones such abscisic acid (ABA), auxin (AUX), gibberellic acid (GA), and ethylene (ET) (Rahoui et al., 2010; Vijayaragavan et al., 2011). Cd toxicity also induces the upregulation of glutathione peroxidase (Gpx) expression and decreases the activity of glutathione reductase (Branca et al., 2020). Additionally, Cd toxicity hinders the proper functioning of mitochondria (Genchi et al., 2020).

On the other hand, Co triggers DNA methylation in *Vicia faba* seeds (Rancelis et al., 2012) while Cu is harmful to young sunflower plants, causing oxidative stress by producing ROS and reducing catalase activity (García et al., 1999; Pena et al., 2011). Under stress conditions, the germination rate is decreased and there is a stimulation of biomass mobilization, which hinders the breakdown of starch and sucrose in reserve tissue (Sethy and Ghosh, 2013).

Cu poisoning induces oxidative stress by increasing the expression of antioxidant and stress-related proteins, hence altering metabolic processes (Liu et al., 2020). Ni is a noxious agent that impacts plant species by altering enzyme function and hindering seed germination and growth (Pandolfini et al., 2006; Sethy and Ghosh, 2013). It impacts the process of breaking down and moving food reserves in plants, resulting in decreased plant height, root length, fresh and dry weight, chlorophyll content, enzyme carbonic anhydrase activity, malondialdehyde content, electrolyte leakage, and photosynthetic pigments (Alam et al., 2007). Ni stress has a detrimental impact on *Brassica nigra* seeds, resulting in a substantial decrease in growth, leaf water potential, pigments, and photosynthetic machinery (Yusuf et al., 2012).

## 5 The beneficial roles of metals in plants

Plants need six HMs, namely, Cu, Zn, Mn, Mo, Fe, and possibly Ni. Cu is a metallic element that is essential for the process of photosynthesis and is present in numerous enzyme systems (Festa and Thiele, 2011). Cu also improves the flavor and color of fruits, vegetables, and flowers by increasing the sugar content in plants (López-Vargas et al., 2018). Furthermore, Cu is essential for the respiration of plants and is involved in the production and formation of seeds (Chen et al., 2022). Zn, on the other hand, is a component of the enzymatic system and plant metabolism (Hamzah Saleem et al., 2022). It is essential for the synthesis of RNA and protein as well as the production of chlorophyll and carbohydrates (Umair Hassan et al., 2020; Costa et al., 2023). Additionally, it is involved in the production of growth hormones, which are responsible for the regulation of plant growth and stem elongation (Saboor et al., 2021).

In addition, Zn permits plants to endure frigid temperatures (Kudo et al., 2023). Mn is essential for photosynthesis and respiration (Alejandro et al., 2020). The availability of N, P, and Ca to the plant is enhanced by Mn, which facilitates their decomposition (Yang et al., 2021b). Additionally, Mn activates numerous enzyme systems, some of which are responsible for safeguarding plants from specific environmental stressors, such as drought, winter cold, salt damage, and ozone damage as well as specific soil-borne diseases and fungal leaf diseases (Alejandro et al., 2020). Additionally, it facilitates pollen tube development and pollen germination (Sawidis et al., 2021). It is also essential for the production of chlorophyll and protein (Mousavi et al., 2011).

**Figure 2:**
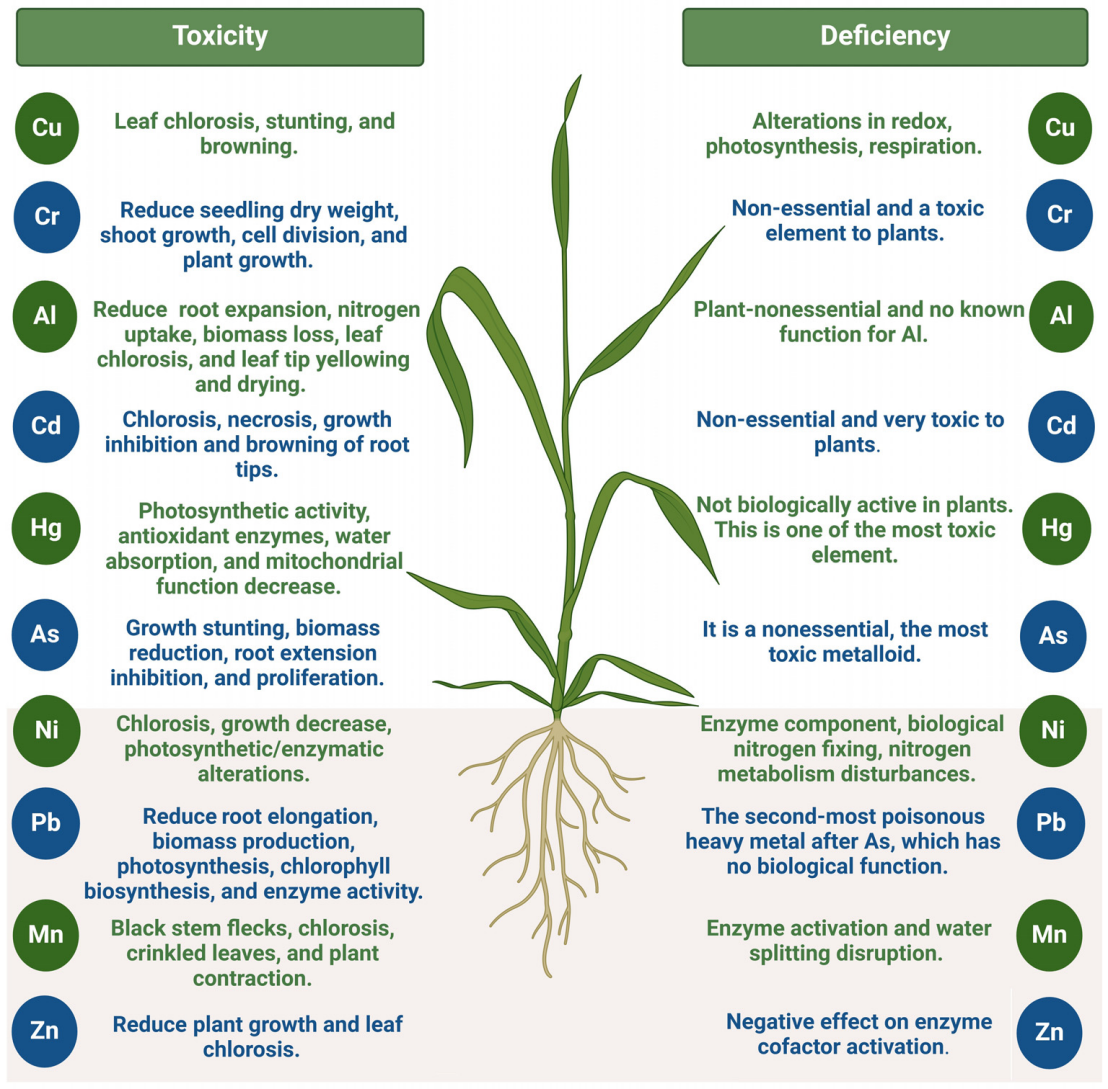
Toxic and inefficient impact of heavy metals on plants. This figure was made using BioRender.

Conversely, the plant necessitates Mo to convert nitrates into ammonia, a form that it can assimilate (Liu et al., 2022). Mo is also indispensable for specific microorganisms, such as rhizobia, which have a symbiotic relationship with legumes and contribute to the fixation of atmospheric N in legumes (Bursakov et al., 2023). Mo also facilitates the conversion of inorganic forms of phosphorus into organic forms that are able to be absorbed by the plant (Seeda et al., 2020). In contrast, Fe is critical to the plant’s development and health. It is vital in metabolic activities such as DNA synthesis, energy transmission, photosynthesis, and respiration (Rai et al., 2021; Ning et al., 2023). Finally, Ni is essential for the biological fixation of atmospheric N in legumes and the metabolism of N in plants (Mendes et al., 2023). It is involved in the metabolism, iron assimilation, senescence, and disease resistance of plants (Begum et al., 2022). The beneficial effects and toxicity of critical HMs in various plants are reviewed in **Figure 2**.

Metal ions can cause hormetic reactions in plants (Salinitro et al., 2021). Hormesis is a biphasic reaction to diverse chemicals in living organisms that likely produce an adaptive stress response (Mattson, 2008). It is likely an adaptive stress response induced by a disturbance of homeostasis caused by low levels of biotic or abiotic stimuli (Vargas-Hernandez et al., 2017). HMs stimulate plant hormetic responses, which may impact nutrient absorption, activate particular defense processes, create ROS, activate antioxidant responses, and improve photosynthetic system efficiency, leading to increased biomass. This mechanism is thought to be an adaptive reaction to stress (Salinitro et al., 2021).

On the other hand, plants that efficiently protect themselves against one kind of stress may increase their tolerance to other types of stress (Perincherry et al., 2021). Cross-tolerance is a phenomena that highlights plants’ capacity to swiftly adjust to changing environments, exhibiting their well-developed and durable defensive regulatory networks (Foyer et al., 2016). HMs may endanger herbivores, and HMs have been shown in studies to have an effect on fungi and insects, particularly aphids (Chen et al., 2020a). Air and soil pollution, especially HMs, may alter plant–insect or plant–disease connections. This pollution may be harmful, causing a hormesis effect and influencing these species’ behavior and metabolism (Perincherry et al., 2021).

## 6 HMs–Transporters in plants

Metal ions are transported by a diverse array of transporters in organelles. Recent advancements in molecular and genetics research have found many crucial gene families that play a role in metal transport (Drew et al., 2021) (**Table 1**). These gene families have the potential to greatly contribute to HMs’ tolerance in hyperaccumulator plants. So far, scientists have discovered many different types of metal transporter proteins in plants (Singh et al., 2015). These transporters are responsible for the safe storage of ions and the assistance of plants in recovering from the adverse effects of metal stress. These proteins have important roles in absorbing, transporting, sequestering, and storing metals in particular parts of the cell. Additionally, they have a noteworthy impact on the regulation of metal levels inside plant cells (Drew et al., 2021).

**Table 1:** List of gene families that play a role in metal transport.

Gene family	Genes	Plant	Roles	References
ZIP	*AtIRT1*, A*tIRT2*, and *AtIRT3*	*Arabidopsis thaliana*	*AtIRT1* is involved in the uptake of vital metals including iron, zinc, manganese, nickel, and cobalt as well as non-essential elements such as cadmium from the soil into the cells of the root epidermis. *AtIRT2* has a role in the segregation of iron and zinc into internal storage vesicles to mitigate the harmful effects of excessive metal levels. *AtIRT3* promotes the absorption of iron and zinc but does not improve the uptake of cadmium and manganese	(Grotz et al., 1998; Guerinot, 2000)
ZIP	*OsIRT1* and *OsIRT2*	*Oryza sativa*	They enhance the absorption of iron, zinc, and cadmium	(Nakanishi et al., 2006)
ZIP	*ZmIRT1*	*Zea mays*	It has a role in the transportation of iron and zinc	(Li et al., 2015)
ZIP	*NcZNT1*	*Nicotiana caerulescens*	It is a critical zinc-regulated, iron-regulated transporter-like proteins that is implicated in the hyper-accumulation and tolerance of zinc and cadmium. It is also responsible for the translocation of metals to the shoot through xylem-mediated processes	(Lin et al., 2016)
ZIP	*AtZIP4*	*Arabidopsis thaliana*	It is accountable for the translocation of metals to the shoot through xylem-mediated mechanisms	(Lin et al., 2014, Lin et al., 2016)
ZIP	*IRT3, ZIP3, ZIP6, ZIP9*, and *ZIP12*	*Arabidopsis halleri*	They ensure the homeostasis of heavy metals in the entirety of plants	(Chiang et al., 2006)
CDF	*ZAT1* (*MTP1*)	*Arabidopsis thaliana*	The primary expression of ZAT is found throughout the plant, and its expression is augmented by an increase in zinc concentration	(van der Zaal et al., 1999)
CDF	*AtMTP1* and *AtMTP3*	*Arabidopsis thaliana*	They are accountable for the vacuolar transport of zinc	(Kobae et al., 2004; Desbrosses-Fonrouge et al., 2005; Arrivault et al., 2006)
CDF	*AhMTP1-A1* and *AhMTP1-A2*	*Arabidopsis halleri*	Under conditions of excessive zinc stress, they were upregulated in the roots	(Dräger et al., 2004; Shahzad et al., 2010)
CDF	*AhMTP1-B1*	*Arabidopsis halleri*	It manifested in roots when zinc deficiency was present	(Shahzad et al., 2010)
CDF	*AhMTP1-C* and *AhMTP1-D*	*Arabidopsis halleri*	Under conditions of excessive zinc stress, they were downregulated in the shoot and roots	(Shahzad et al., 2010)
CDF	*AtMTP11*	*Arabidopsis thaliana*	It is upregulated in the presence of a zinc deficiency	(Delhaize et al., 2007; Peiter et al., 2007)
CDF	*NtMTP1-A* and *NtMTP1-B*	*Nicotiana tabacum*	They upregulate in response to deficiencies in zinc and copper	(Shingu et al., 2005; Ricachenevsky et al., 2013)
CDF	*OsMTP1*	*Oryza sativa*	It is upregulated in the shoot and root in the presence of an excess of zinc, cobalt, nickel, cadmium, and iron	(Yuan et al., 2012; Menguer et al., 2013)
CDF	*PtMTP11.1* and *PtMTP11.2*	*Populus trichocarpa*	They upregulate under manganese deficiency	(Peiter et al., 2007)
COPT	*COPT1*, *COPT2*, and *COPT6*	*Arabidopsis* *thaliana*	They are indispensable elements of the primary pathway for cellular high-affinity uptake of copper	(Sancenón et al., 2003; Yuan et al., 2011; Garcia-Molina et al., 2013)
COPT	*COPY2*	*Arabidopsis* *thaliana*	This is a cell surface transporter that is primarily found in all regions of plants, with a particular abundance in roots, young leaves, apical meristems, trichomes, and anthers	(Perea-García et al., 2013)
COPT	*COPT5*	*Arabidopsis* *thaliana*	It is thought to have a role in maintaining balance inside cells	(Garcia-Molina et al., 2011)
ABC	*AtABCC1* and *AtABCC2*	*Arabidopsis* *thaliana*	They have been linked via vacuolar sequestration to phytochelatin-mediated cadmium and mercury detoxification	(Park et al., 2012)
ABC	*OsABCB14*	*Oryza sativa*	It is shown to be in charge of iron homeostasis	(Xu et al., 2014)
ABC	*OsABCG43*/PDR5	*Oryza sativa*	Induced in rice roots during cadmium stress, it may be implicated in cadmium detoxification by compartmentalizing cadmium into organelles	(Oda et al., 2011; Xu et al., 2014)

Metal transporters can be classified into six main groups: natural resistant-associated macrophage protein (NRAMP) (Li et al., 2024), Zn-regulated, Fe-regulated transporter-like proteins (ZIP) (Shuting et al., 2022), cation diffusion facilitator (CDF) transporters (Kolaj-Robin et al., 2015; El-Sappah et al., 2021b; El-Sappah et al., 2023), yellow stripe-like (YSL) proteins (Islam et al., 2020), and P1B-type HMs ATPases (HMAs) (Batool et al., 2023).

Cell organelles include specialized compartments dedicated to certain processes, including photosynthesis, respiration, phytohormone production, and metal detoxification (Jogawat et al., 2021). Among these transporters, the vacuole plays a crucial role in the accumulation and compartmentalization of metals for their detoxification. This is a significant strategy for lowering metal stress-related ailments in plants (Emamverdian et al., 2015; Sharma et al., 2016). Chloroplasts and mitochondria need transition metals to carry out essential operations such as photosynthesis, electron transport system, photoprotection, and other processes. Maintaining metal homeostasis is crucial for the optimal functioning and structural integrity of chloroplasts and mitochondria (Nouet et al., 2011).

Metal and nonmetal ions are primarily stored in vacuoles within plant cells. Additionally, the neutralization and mitigation of the detrimental effects of metal ions are contingent upon their storage in the vacuole (Hall, 2002). Tonoplasts (vacuolar membrane) contain a variety of transport proteins, such as MTPs, ABC transporters (ABCCs, ABCGs), HMAs, Ca^2+^ exchangers (CAXs), and NRAMPs. These proteins either remove metals from the cytosol or deposit them in the vacuolar area (Zhang et al., 2018a).

Chloroplasts have a crucial role in hosting transition metals due to their involvement in the process of photosynthesis and the breakdown of water molecules (Schmidt et al., 2020). Chloroplasts play a crucial role in reducing the harmful effects of metal toxicity by capturing metal ions to facilitate metal detoxification. The double membrane structure of the cellular envelope allows for the protection of its integrity and the detoxification of metals in the intermembrane gap. Various transporters located on the inner and outer membranes are necessary to maintain the homeostasis of chloroplastic metals (Nouet et al., 2011).

Mitochondria are a crucial cellular organelle occasionally referred to as the cell’s powerhouse due to their substantial involvement in chemosmosis. This is because certain metals are essential for the correct functioning of mitochondria, as they serve as cofactors for critical enzymes and are also involved in the composition of electron transport molecules (Jogawat et al., 2021). Therefore, it is imperative to maintain the equilibrium of metals within the mitochondria. ATMs and mitochondrial iron transporters (MITs) are the two primary categories of transporters that modulate metal levels in mitochondria (Nouet et al., 2011).

Golgi apparatus is an essential component of the endomembrane system, which plays a crucial role in directing membrane-bound proteins (such as transporters) to either the plasma membrane or organelles (Jogawat et al., 2021). The major purpose of this is to regulate the balance of metals in the body. Metal transporters are also found in the Golgi apparatus to detect and import metals for their arrangement (Bressler et al., 2007). When exposed to metal stress, the Golgi apparatus system responds by reorganizing the endomembrane system and storing excess metals in vesicles (De Caroli et al., 2020). This process involves reducing the number of metal transporters at the plasma membrane. This substantially reduces the detrimental impacts of metals (De Caroli et al., 2020).

Finally, endoplasmic reticulum (ER) is also part of the endomembrane system and has a vast lumen to store and utilize metals for different purposes (De Caroli et al., 2020). Broad-specificity transporters for Cd, Cu, and Zn are identified on the ER membrane and are sometimes found to be localized on the plasma membrane. This common localization might be due to the continuum of the endomembrane system as part of the secretory pathway (Jogawat et al., 2021).

## 7 Molecular, biochemical, and metabolic plant responses toward HMs

### 7.1 Negative impact of metals on plants

Exposure to HMs causes several reactions in plants, including physiological, biochemical, and agricultural production responses (Singh et al., 2020). The toxicity of HMs is influenced by a variety of factors, such as the plant species, the concentration of the individual metal, its chemical structure, soil composition, and pH level (Abd Elnabi et al., 2023). Certain HMs, including Cu and Zn, are essential for the vegetative plant growth (Arif et al., 2016). HMs can participate in enzyme processes by forming complexes with enzymes and substrates, acting as cofactors and activators (Witkowska et al., 2021). Trace metal nutrients have a vital role in redox reactions, electron transportation, and structural functions in nucleic acid processing (Sunda, 2012).

Additionally, HMs have various effects on the functioning of the photosynthetic system at different levels of organization (Ventrella et al., 2009). HMs directly affect plants by interfering with the PS I and PS II processes and indirectly affect photosynthesis, growth, and yield (Singh et al., 2015). Additionally, certain HMs, including Cd and Hg, possess phytotoxic properties that impede metal-sensitive enzymes, resulting in growth retardation and the mortality of organisms (Alengebawy et al., 2021). HMs can be classified into two categories based on their ability to undertake redox reactions: redox-active and redox-inactive (Ercal et al., 2001). The redox reaction within cells is facilitated by redox-active transition metals, including Fe, Cu, Cr, and Co (Kostenkova et al., 2022). This process leads to the production of superoxide (O_2_^•−^), hydrogen peroxide (H_2_O_2_), and hydroxyl radicals (•OH) (Collin, 2019). The oxidative stress is induced by the indirect interactions with the antioxidant defense system, disruption of the electron transport chain, and induction of lipid peroxidation, which are the results of exposure to redox-inactive HMs (Bhattacharyya et al., 2014).

In plants, HMs are involved in the production and release of free radicals through chemical reactions, metabolic pathways, and physiological processes (Emamverdian et al., 2015). The ROS are generated by biological systems through the production of radicals that are centered on oxygen, S, N, and carbon (Phaniendra et al., 2015). The lipid content of thylakoid membranes is altered in plants that are exposed to HMs stress, which leads to membrane degradation and lipid peroxidation (Emamverdian et al., 2015). The primary site of lipid peroxidation is polyunsaturated fatty acids, and it is comprised of three distinct phases: initiation, advancement, and termination. The functionality of PS II is impeded by HMs, which leads to an increase in the formation of O_2_^•−^ in leaves and an increase in lipid peroxidation (Hasanuzzaman et al., 2020). Recent research has revealed that HMs can damage numerous physiological systems by generating ROS that induce lipid peroxidation (Shahid et al., 2014). Furthermore, the rate of photosynthesis and PS II can be significantly affected by the by-products of lipid peroxidation (Pospíšil and Yamamoto, 2017).

Chlorophylls (Chl) and carotenoids are essential pigments that are involved in the conversion of solar energy to chemical energy during photosynthesis (Hashimoto et al., 2016). The production of photosynthetic compounds is specifically influenced by HMs (Ventrella et al., 2009). Chlorosis and plant growth retardation are frequently observed in metal-contaminated environments. These findings suggest that the biosynthesis of photosynthetic compounds has been disrupted (Yadav, 2010). Consequently, these variables influence the proliferation of plastids, the efficiency of photosynthesis, and the overall metabolism. Additionally, HMs inhibit the accumulation of photosynthetic compounds (Ventrella et al., 2009).

### 7.2 The mechanism of HM uptake and tolerance

The development of plants as phytoremediation agents is contingent upon an understanding of the genetic basis and interrelated network of physiological and molecular mechanisms that govern plant tolerance to specific HMs (Hossain et al., 2012). Different plant species may have developed distinct mechanisms to tolerate excessive HMs, and even within a single plant species, multiple mechanisms may be in operation (Asiminicesei et al., 2024). To endure excessive HMs, plants possess both constitutive and adaptive mechanisms (Hasanuzzaman et al., 2013). To identify the underlying mechanisms of HMs’ accumulation, tolerance, and adaptive mechanisms to contend with HM stress, physiological, biochemical, and molecular approaches are still being employed (Mashabela et al., 2023).

**Figure 3:**
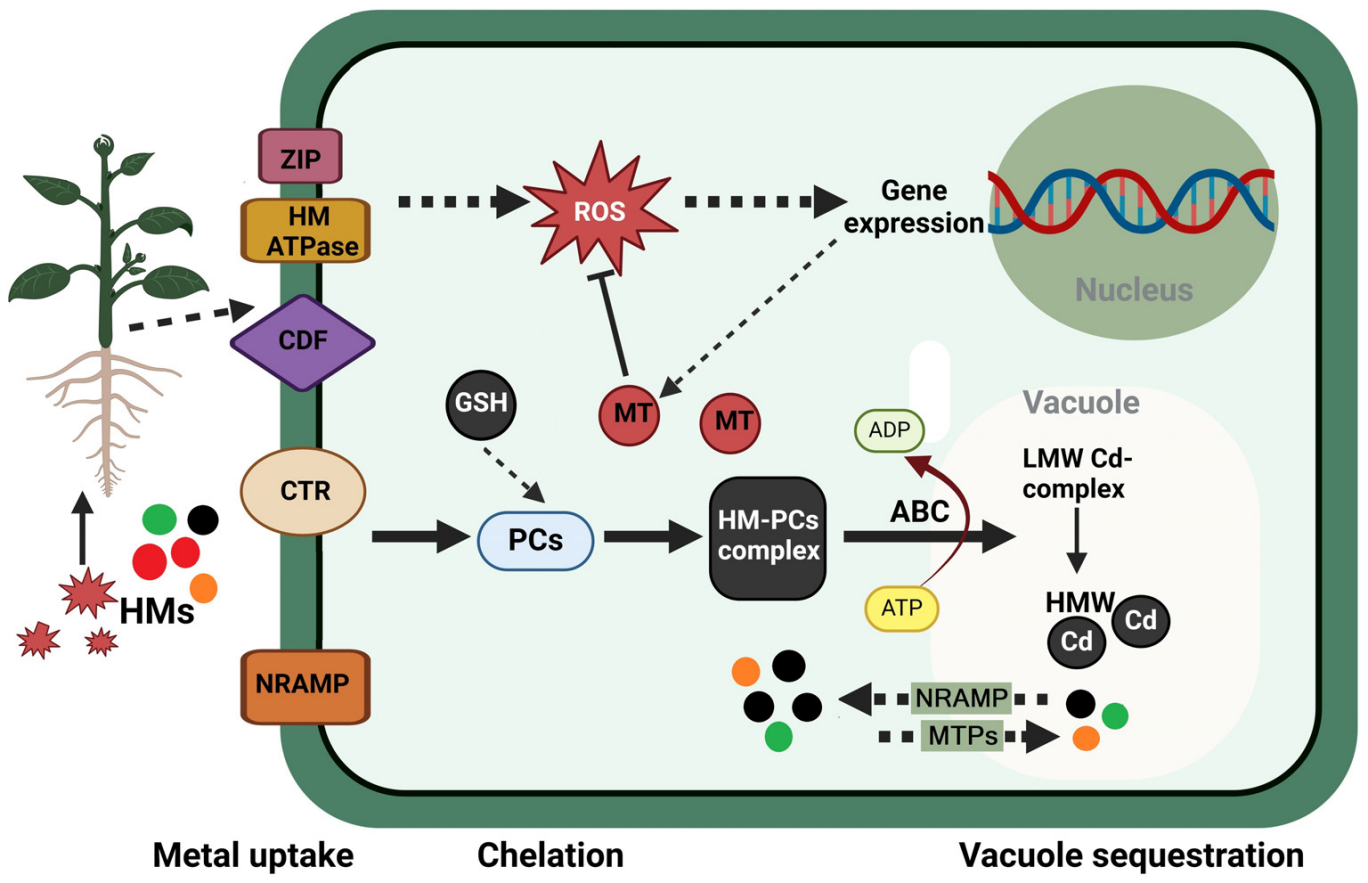
Sequestration of heavy metals in plant cells within the vacuoles. The uptake of heavy metal (HMs) ions is facilitated by a variety of transporters, such as the cation diffusion facilitator (CDF) family, the heavy-metal-transporting ATPase (HM ATPase), the copper transporter (CTR), the zinc-regulated, iron-regulated transporter-like proteins (ZIP), and the natural resistance-associated macrophage protein (NRAMP). For instance, HMs, such as Cd^2+^, enter the cytosol and initiate the production of phytochelatins (PCs) after being transported by members of the ZIP family. PCs are produced through a transpeptidation reaction from reduced glutathione (GSH) in a non-translational manner. The primary function of PCs is to bind cytosolic HMs, which results in the formation of the HM–PC complex. In the case of Cd^2+^ ions, these bind with low-molecular-weight (LMW) complex and form the LMW Cd-complex. This complex is subsequently transported into the vacuole by a tonoplast-localized ATP-binding-cassette (ABC) transporter. The LMW Cd-complex is collected and converted into a high-molecular-weight (HMW) complex within the vacuole. This complex contains supplementary Cd^2+^ ions. The tonoplast-localized cation/proton exchanger (CAX) transporters facilitate the direct interaction between these HMW complexes and protons, thereby enabling them to access the vacuole. Metal tolerance proteins (MTPs) and NRAMPs are transporters that are present in the tonoplast. They are accountable for the migration of metal ions to facilitate compartmentalization or remobilization. Organic acids, amino acids, and metallothioneins (MTs) are among the chelators that contribute to the regulation of metal levels in the cytosol to a safe and low level. ROS, reactive oxygen species. This figure was made using BioRender.

Among the adaptive mechanisms that tolerant plants have evolved are the synthesis of particular phytochelatins (PCs) and metallothioneins (MTs), induction of mechanisms opposing the effects of ROS and MG, induction of stress proteins, the biosynthesis of proline (Pro), polyamines (PAs), and signaling molecules like salicylic acid (SA) and nitric oxide (NO) (Hossain et al., 2012; Hasanuzzaman et al., 2019). **Figure 3** demonstrates the process of HMs’ sequestration in plant cells, specifically within the vacuoles. HMs are absorbed by plants through root interception, entrance into roots, and translocation to the stem (Khan et al., 2023). The entrance of HMs into the organism is contingent upon the sort of HM (Yimer et al., 2024).

The prevention of superfluous HMs from infiltrating the plant is one method of reducing or preventing the toxic effects of HMs. Plants can accomplish this by precipitating or complexing HMs in the root environment (Riyazuddin et al., 2021). Plants can precipitate HMs by either increasing the pH of the rhizosphere or excreting anions, such as phosphate (Hinsinger et al., 2003; Chen et al., 2017). In response to Al stress, root exudation of phosphate has been observed in maize (Calderón-Vázquez et al., 2011). Additionally, malate exudation from the roots of sorghum and citrate exudation from the roots of maize have also been documented in response to Cd stress (Piñeros et al., 2002). These results lend credence to the hypothesis that the HM-binding capabilities of root exudates may serve as a critical mechanism for stabilizing HMs in the vicinity of the root, thereby rendering them unavailable to the plant and reducing the toxicity encountered by the plant (Hossain et al., 2012).

There must also be other processes since certain tolerant and hyperaccumulator plants really absorb more HMs than sensitive plants. An essential adaptive strategy for HM tolerance in plants is the cellular exclusion of HMs (Riyazuddin et al., 2021). The apoplastic space is the location of a significant proportion of HMs in plant roots, which implies an exclusion mechanism (Sattelmacher, 2001). The cell wall–plasma membrane interface has the potential to serve as a site of HM tolerance, as it accumulates substantial amounts of HMs (Danouche et al., 2021). The plant cation exchange capacity (CECs) of sensitive wheat cultivars is substantially lower than that of tolerant cultivars (Masion and Bertsch, 1997). This implies that tolerant cultivars utilize a high CEC to complex HMs at the cell wall and obstruct their entry into the cell (Bortoloti and Baron, 2022).

Once HMs infiltrate the cell, plants employ a variety of strategies to mitigate their toxicity (Pande et al., 2022). One approach is to transport or sequester HMs into the vacuole, which serves as an appropriate storage reservoir for excessively accumulated HMs (Peng and Gong, 2014). Vacuolar assimilation of the majority of solutes is stimulated by two vacuolar proton pumps: a vacuolar proton-ATPase (V-ATPase) and a vacuolar proton pyrophosphatase (V-Ppase) (Hossain et al., 2012). Grasses have the capacity to actively transport Zn into vacuoles, with more tolerant clones being able to maintain the process at higher external Zn levels than sensitive clones (Brookes et al., 1981). Either channels or transporters can facilitate uptake. Genetic and molecular techniques have identified a variety of genes (**Table 2**) that are involved in the uptake of transition HM ions into cells, the sequestration of HMs in the vacuole, the remobilization of HMs from the vacuole, the loading of HMs in the xylem, and the discharge of HMs (Gill et al., 2021).

**Table 2:** Compilation of genes exhibiting variable expression in response to various heavy metals.

Plant	Gene	Metal	Response	References
*Vigna radiata*	*irt1*and *irt2*	Cadmium	Treatments involving cadmium under conditions of sufficient iron	(Muneer et al., 2014)
*Arabidopsis thaliana*	*AtABCC3* and *AtABCC6*	Cadmium	The phenomenon of tolerance during seedling development mediated by phytochelatin	(Brunetti et al., 2015)
*Arabidopsis thaliana*	*CAX2* and *CAX4*	Cadmium	It plays a role in the storage of cadmium in the vacuoles, which gives it the ability to tolerate heavy metals	(Koren’Kov et al., 2007; Mei et al., 2009)
*Arabidopsis thaliana*	*AtHMA4*	Cadmium	The expression of the gene decreased when exposed to cadmium stress	(Xu et al., 2010)
*Arabidopsis thaliana*	*AtNHX1*	Cadmium	It is accountable for the storage of metabolites in vacuoles and enhances tolerance	(Yao et al., 2020; Riyazuddin et al., 2021)
*Oryza sativa*	*cadA* and *bmtA*	Cadmium	The accumulation of cadmium and the production of cadmium-nanoparticles have been found to enhance tolerance by reducing oxidative stress	(Shi et al., 2020)
*Nicotiana tabacum*	*TaMT3*	Cadmium	It resulted in an elevation of superoxide dismutase activity and provided tolerance	(Zhou et al., 2014)
*Hibiscus cannabinus* L.	*WRKY*, *GRAS*, *MYB*, *bHLH*, *ZFP*, *ERF*, and *NAC*	Cadmium	The molecular mechanism underlying enhanced tolerance	(Chen et al., 2020b)
*Fragaria vesca*	*FvABCC11*	Cadmium	Enhancement of tolerance by the utilization of ATP binding cassette (ABC) transporters	(Shi et al., 2020)
*Brassica napus*	*BnaABCC3* and *BnaABCC4*	Cadmium	Augmentation of stress tolerance	(Zhang et al., 2018b)
*Triticum aestivum*	*TaABCC*	Cadmium	Unique molecular manifestation and heightened resilience	(Bhati et al., 2015)
*Oryza sativa*	*OsHMA3* and *OsABCC9*	Cadmium	Participating in the study of plant cadmium tolerance	(Sasaki et al., 2014; Yang et al., 2021a)
*Oryza sativa*	*OsMYB45, OsCATA* and *OsCATC*	Cadmium	*OsMYB45* induces upregulation of *OsCATA* and *OsCATC* receptors, enhances catalase activity in plants, and reduces rice’s susceptibility to cadmium	(Hu et al., 2017)
*Populus alba*	*PyWRKY75*	Cadmium	The upregulation of *PyWRKY75* resulted in enhanced tolerance to cadmium	(Wu et al., 2022)
*Oryza sativa*	*OsNAC15,OsZIP7* and *OsZIP10*	Cadmium	The regulation of zinc and cadmium tolerance is mediated by *OsNAC15* by its interaction with the ZDRE motif located in the promoters of *OsZIP7* and *OsZIP10*, resulting in the inhibition of their transcription	(Zhan et al., 2022)
*Arabidopsis thaliana*	*MAN3*	Cadmium	The glutathione-dependent pathway is responsible for the regulation of cadmium tolerance	(Chen et al., 2015)
*Arabidopsis thaliana*	*AtFC1*	Cadmium	The control of both antioxidants and antioxidant enzymes is attributed to several factors	(Hossain et al., 2012)
*Arabidopsis thaliana*	*MYB40* and *PCS1*	Arsenic	The expression of *PHT1;1* was directly suppressed to decrease the absorption of As(V) into plant cells, while the expression of *PCS1* was directly increased to increase the amount of PCs, which formed complexes with As(III). The expression of *PHT1;1* was suppressed, while the expression of *PCS1* was upregulated	(Sung et al., 2009; Castrillo et al., 2013)
*Triticum aestivum*	*TaCATs*	Arsenic	Stress tolerance	(Tyagi et al., 2021)
*Oryza sativa*	*OsABCC1*	Arsenic	Sequestration can manifest in various parts of rice plants, including roots, stems, leaves, and husks. The presence of vacuoles plays a crucial role in mitigating the distribution of arsenic within rice grains	(Song et al., 2014)
*Oryza sativa*	*OsLsi1* and *OsLsi2*	Arsenic	They can lead to an augmentation in the absorption of arsenate by roots	(Pan et al., 2020a)
*Oryza sativa*	*OsLsi3/OsLsi6*	Arsenic	They result in a significant rise in the buildup of arsenic in shoots during the stages of heading to milk	(Pan et al., 2020a)
*Arabidopsis thaliana*	*ATQ1*	Arsenic	ATQ1 deficiency results in a reduced outflow of arsenic in roots and a notable rise in arsenic accumulation in shoots	(Chao et al., 2017)
*Oryza sativa*	*CCoAOMT*	Copper	Increased lignin synthesis and improved tolerance	(Su et al., 2020)
*Nicotiana tabacum*	*EhMT1*	Copper	It results in reduced hydrogen peroxide production and enhanced tolerance	(Xia et al., 2012)
*Arabidopsis thaliana*	*MT2a* and *MT3*	Copper	They are significantly stimulated by copper exclusively in the root tips and young leaves	(Guo et al., 2003)
*Imperata cylindrica*	*CRK10*, *SDI1, PHO1*, *PHT1-11*, *VIT1*, *VTC2*, *PAE7*, *SWEET3*, and *REX4*	Copper	Genes that are expressed differently in shoots under situations of copper stress	(Vidal et al., 2021)
*Imperata cylindrica*	*Mn-SODs*, *SOD1*, *ATOX1*, *HEPHL1*, *HMA5*, *NBR1*, *ACT1*, *Act87E*, *Arp2*, and *Actobindin-A*	Copper	These genes exhibit a correlation with copper-tolerant systems in roots	(Vidal et al., 2021)
*Jatropha curcas*	*JcMT2a* and *JcPAL*	Lead	The process of antioxidant accumulation, such as the presence of flavonoids and phenolics, as well as metal detoxification	(Pan et al., 2020b)
*Nicotiana tabacum*	*tCBP4*	Lead	Increased tolerance	(Sunkar et al., 2000)
*Arabidopsis thaliana*	*ACBP1*	Lead	Increased gene expression and improved tolerance	(Xiao et al., 2008; Du et al., 2015)
*Linum usitatissimum*	*LuACBP1* and *LuACBP2*	Lead	The transcript level was elevated in the transgenic group, resulting in enhanced tolerance	(Pan et al., 2020b)
*Medicago sativa*	Sucrose synthase, P5CS, and δ-OAT	Lead	Lead resistance	(Wang et al., 2021b)
*Medicago sativa*	YUCCA, 4CL, CCR, F5H, and COMT	Lead	Their increased expression results in the growth of roots during lead-induced stress	(Wang et al., 2021b)
*Medicago sativa*	NRAMP, MATE, HIPPs, MTP, and ABC transporter	Lead	They were subjected to lead stress	(Wang et al., 2021b)
*Pogonatherum crinitum*	*CAT*, *SOD*, and *POD*	Lead	Their antioxidant enzyme activities are consistent with the evolving trend of roots	(Zhu et al., 2022)
*Oryza sativa*	*OsSTAR1* and *OsSTAR2*	Aluminum	Reduced concentration of aluminum in the cell wall and increased tolerance	(Huang et al., 2021)
*Arabidopsis thaliana*	*AtALMT1* and *STOP1*	Aluminum	The transcription factor *STOP1t* plays a crucial role in the regulation of *ALMT1* expression, which is essential for the development of aluminum tolerance	(Daspute et al., 2017)
*Arabidopsis thaliana*	*AtBCB*	Aluminum	It provided a certain level of resistance to aluminum	(Ezaki et al., 2000)
*Nicotiana tabacum*	*parB*, *NtPox* and *NtGDI1*	Aluminum	It provided a certain level of resistance to aluminum	(Ezaki et al., 2000)
*Oryza sativa*	*ART1*, *Nrat1*, *OsFRDL4*, *OsALS1*, *OsMGT1*, *ASR5* and *ART2*	Aluminum	They have crucial functions in the resistance to aluminum poisoning	(Bhattacharjee et al., 2023)

Zn-regulated transporter (ZRT), Fe-regulated transporter (IRT)-like protein ZIP family, ATP-binding cassette (ABC) transporters, the P-type metal ATPases, NRAMP family, multidrug resistance-associated proteins (MRP), CDF family of proteins, copper transporter (COPT) family proteins, pleiotropic drug resistance (PDR) transporters, YSL transporter, and CAX are among the well-characterized HM transporter proteins (Toyoda et al., 2008; Pittman and Hirschi, 2016; Wang et al., 2021a; Pacheco et al., 2023). MTs and PCs are two forms of peptide metal-binding ligands that are essential for the detoxification and tolerance of HMs in plants that are subjected to HM stress (Faizan et al., 2024).

PCs are synthesized from GSH and are induced by a variety of HMs, including Cd, Hg, Ag, Cu, Ni, Au, Pb, As, and Zn (Faizan et al., 2024). The activity of ABC transporters accumulates them in the vacuole, thereby restricting the circulation of free Cd^2+^ within the cytosol, and they complex Cd ions through the thiolic group (–SH) of cysteine (Salbitani et al., 2023). PCs are produced by both HM-resistant and HM-sensitive plants; however, certain reports have concluded that PCs are not the primary cause of the hyperaccumulation of Zn, Ni, or Pb (Hossain et al., 2012). The chelation of HM ions is not the sole mechanism of the HM detoxification process (Gulcin and Alwasel, 2022). The HM ion complex is transported to the vacuole and stabilized, thereby forming a complex with sulfides or organic acid, following the activation of PC synthase by HM ions and HM chelation by the synthesized PCs (Faizan et al., 2024). Nevertheless, the HM specificity or species specificity of hyperaccumulation is not adequately elucidated by the formation of HM complexes. Consequently, the precise function of PCs in the HM tolerance mechanism at the cellular level is yet to be ascertained (Emamverdian et al., 2015).

Plant MTs are polypeptides that are cysteine-rich, low molecular weight, and capable of engaging HMs through their cysteine residues (Freisinger, 2011). Their physiological functions encompass the protection against intracellular oxidative damage, the sequestration of toxic HMs, and the maintenance of essential transition HM homeostasis (Subramanian Vignesh and Deepe, 2017). The cysteine residue arrangement has resulted in the division of plant MTs into three classes, which are diverse (Guo et al., 2003). The organization of cysteine residues confers distinct MT isoforms and their capacity to bind and sequester distinct HM ions for the purposes of detoxification and homeostasis (Ruttkay-Nedecky et al., 2013).

Factors such as hormones, cytotoxic agents, and HMs induce MT biosynthesis, which is regulated at the transcriptional level (Thirumoorthy et al., 2007). Gene expression studies have demonstrated that MT genes are differentially regulated in response to a variety of HM stresses (Qu et al., 2024). The role of MTs in HM detoxification and homeostasis has been demonstrated by a variety of data (Ruttkay-Nedecky et al., 2013). However, the metal-inducibility of plant MTs has not always been demonstrated. Additional information regarding the structures and properties of MTs could provide a more comprehensive understanding of their functions and mechanism(s) of action (Hossain et al., 2012). The molecular mechanisms of HM transport, trafficking, tolerance, and homeostasis in plants are likely to be further elucidated through the use of a model system and a model hyperaccumulator, such as *Arabidopsis halleri* and particularly *Thlaspi* species (Pasricha et al., 2021).

In plants, metal chelation can be classified into two categories: internal tolerance and external exclusion. During the external detoxification process, organic acids expelled from plant roots may combine with HM ions to create stable HM–ligand complexes, which may change the HM ions’ mobility and bioavailability (Sabreena et al., 2022). This obstructs the entry of HM ions into plants and prevents their accumulation in sensitive root sites. Organic acids may chelate with HM in the cytosol during internal HM detoxification, resulting in the transformation of the ions into a less toxic or nontoxic form (Gasic and Korban, 2006). Plants generate a variety of ligands for Al, Cd, Cu, Ni, Co, and Zn. Potential ligands for HMs include carboxylic and amino acids, including citrate, malate, and oxalate, histidine and nicotianamine, and phosphate derivatives (phytate), which are involved in detoxification and tolerance (Hossain et al., 2012). Citrate has a significant affinity for chelating HM ions, and other HMs, including Cd, Ni, Co, and Zn, also exhibit a high affinity for citrate (Gasic and Korban, 2006; Hossain et al., 2012).

At low Cd concentrations, citric acid is a significant ligand and contributes to the accumulation and tolerance of Zn (Najeeb et al., 2011). HMs such as Al are also detoxified, and oxalate is secreted by the roots (Zheng et al., 2005). In response to Al stress, buckwheat (*Fagopyrum esculentum* Moench.) secretes oxalic acid from the roots and accumulates nontoxic-Al-oxalate in the leaves (Feng Ma et al., 1998). Consequently, detoxification occurs both internally and externally (Hall, 2002). Histidine and nicotianamine are also involved in the chelation of HM ions in the xylem fluid and within plant cells (Zhakypbek et al., 2024). Nicotianamine is a nonproteinogenic amino acid that is mobile within the plant and has been identified in phloem fluid as well as in root and leaf cells (Klatte et al., 2009). It is suggested that it may be involved in the regulation of HM transfer within plant cells (Takahashi et al., 2003).

Plants that are HM-tolerant frequently prevent HMs from being transmitted from root to stem by either detoxifying them through chelation or storage or retaining them in root cells (Singh et al., 2015). Nevertheless, a unique group of plants known as hyperaccumulators effectively transport HMs to the shoot through the xylem, a process that is likely facilitated by transpiration. They are capable of accumulating HMs from modest external concentrations, with the majority of them being translocated to the shoot (Yang et al., 2005). Hyperaccumulators exhibit an unusually high uptake of HM at the root membrane level, which may be attributed to the presence of a high expression of an HM transporter in the plasma membrane (Skuza et al., 2022). Efficient intracellular compartmentalization and chelation may be the cause of this high HM tolerance.

A complex network of biochemical adaptive strategies, known as the antioxidant system, is present in plants to detoxify a variety of ROS (Dumanović et al., 2020). In general, this system can be divided into two categories. The first group comprises enzymes, including superoxide dismutase, catalase, ascorbate peroxidase, and glutathione reductase, that eliminate oxygen radicals and their metabolites (Rajput et al., 2021). Nonenzymatic compounds such as glutathione, ascorbate, and phenolics comprise the second group. These compounds have the ability to neutralize ROS without transforming into deleterious radicals themselves (Zandi and Schnug, 2022).

The presence of the two HMA proteins, hma2/hma4, is essential for the uptake of Cd in the shoot via the xylem (Kraemer, 2009). When cultivated on regular soil, the shoot of the hma2/hma4 double-mutant shows severe signs of Zn shortage, as previously shown (Haydon and Cobbett, 2007). This suggests that both HMA2 and HMA4 are required for the movement of Zn from the roots to the shoots in regular soils (Claus et al., 2013). On the other hand, the heat shock proteins (HSPs) are molecular chaperones that are essential for the protection and repair of proteins under stress conditions as well as for the folding and assembly of proteins (El-Sappah et al., 2017; Abbas et al., 2022). In response to Cd stress, they can enhance the accumulation of large HSPs, such as HSP70, and are induced by transition metals (Zn, Cu, Cd, Hg, Al, and Cr) (Hasan et al., 2017).

Additionally, HSPs can prevent irreversible protein denaturation as a result of oxidative stress or facilitate proteolytic degradation (Kumar et al., 2022). Nevertheless, the extent of their involvement in HM tolerance is still mainly obscure. Enzymes that modify metal oxidation states or facilitate the incorporation of HMs into organic molecules are known as metal-modifying enzymes (Kaczmarek et al., 2009). After being supplied with Cr(VI) in nutrient culture, *Eichhornia crassipes*, a water hyacinth, accumulated innocuous Cr(III) in its root and branch tissues (Giri and Patel, 2011). This implies that *E. crassipes* detoxified Cr(VI) during root assimilation and transported a portion of the detoxified Cr to leaf tissues. A reductase at the root cell membrane in dicots reduces Fe and potentially Cu prior to assimilation (Cohen et al., 1997).

Plants may reduce harmful substances (e.g., HMs) through a process called *in situ* reduction, which can be advantageous for phytoremediation by helping to detoxify the environment (Kafle et al., 2022). Metal-responsive transcription factor 1 (MTF-1) plays a crucial role in the cellular response and tolerance to HM stress by activating genes important for HM uptake, transport, and detoxification (Wang et al., 2004). The TFs involved in the reaction to HM stress and tolerance have been reported in several plant species (Niekerk et al., 2024). On the other hand, oxidative stress and antioxidative defense systems are induced by HM stress (Mansoor et al., 2023). These systems are constituted of free-radical-scavenging molecules, such as ascorbate (AsA) and GSH, and the enzymes involved in their biosynthesis and reduction (Rajput et al., 2021).

During times of stress, specifically HM stress, SA interacts with many plant hormones, including AUX, ABA, and GA, to promote the synthesis of antioxidant chemicals and enzymes. This interaction serves to notify and assist plants treated with HM, helping to alleviate the stress caused by HM (Sharma et al., 2020). SA is a natural signal molecule that is crucial for the regulation of physiological and biochemical processes, thereby enhancing the resistance of plants to biotic and abiotic stresses (Mishra et al., 2024).

On the other hand, Pro accumulation in response to HM stress has also been extensively documented (Hayat et al., 2012). Enhanced protection against Cd stress is provided by increased Pro levels in microalgae (Siripornadulsil et al., 2002). Pro plays a vital role in mitigating the harmful effects of Cd stress by protecting against damage caused by free radicals and maintaining a controlled reducing environment within the cell rather than just isolating Cd (Hayat et al., 2012). However, PAs are organic cations that exist naturally and possess nonenzymatic antioxidant characteristics. They are thought to function as second messengers in regulating plant growth and development processes (Raychaudhuri et al., 2021).

PAs and Pro are components of the “general adaptation syndrome” (GAS) response to environmental adversities, including nutrient scarcity, HMs, and low temperatures (Gill et al., 2012). Engineered plants that overexpress genes involved in the biosynthesis of PAs exhibit an enhanced ability to withstand a range of environmental stressors, including HMs (Kajla et al., 2023). By modulating the level and toxicity of ROS and hormones, NO, a ubiquitous bioactive signaling molecule, serves a critical function in a wide range of physiological processes in plants (Jomova et al., 2023). By governing the general mechanisms for cellular redox homeostasis and promoting the transformation of O_2_^•−^ to H_2_O_2_ and O_2_, NO safeguards plants from oxidation damage (Huang et al., 2019).

NO may also safeguard cells from oxidative processes by promoting the synthesis of GSH (Lu, 2013), in addition to its direct ROS scavenging activity and the modulation of lipid peroxidation through lipoxygenase (LOX) inhibition. Under HM stress, exogenous NO can effectively induce tomato seedlings to modify their physiological and biochemical mechanisms to protect against Cu toxicity, thereby preserving their metabolic capacity and normal growth capabilities (Singh et al., 2015).

## 8 Challenges and prospective

It is crucial to examine the interactions between different HMs in plants. Certain HMs may have synergistic effects, whereby their collective presence amplifies toxicity beyond what would be anticipated based on individual doses (Angon et al., 2024). Conversely, some combinations may have antagonistic effects, where one metal reduces the toxicity of another. In order to assess and control risks, it is important to acknowledge these linkages. Subsequent research should strive to measure the combined impacts of HMs on the physiological processes, growth, and reproductive capabilities of plants. In order to do this, researchers may examine the correlations between dosage and response, the patterns of bioaccumulation, and the effects, particularly on different tissues. There has been a scarcity of research on agricultural genetic diversity and the mechanisms of plant adaptation. The sensitivity and tolerance of plants to HMs might vary depending on the species and genotypes (Emamverdian et al., 2015).

Studying the way various plant species and genotypes react to environmental difficulties might impede the progress of creating metal-tolerant agricultural cultivars. It is crucial to bridge this gap in order to comprehend the ability of plants to withstand challenges, provide guidance for agricultural activities in polluted areas, and assist in environmental remediation efforts. Furthermore, investigating the transfer of HMs to crops is crucial for the development of sustainable agriculture. Gaining insight into the mechanisms by which HMs persist and migrate throughout successive plant generations may provide valuable guidance for addressing pollution in affected areas. This knowledge can be particularly useful in developing phytoremediation systems that effectively eliminate toxins from contaminated soil.

The current scientific investigations have mostly concentrated on the detection and analysis of microplastics in soil. Nevertheless, there is an increasing curiosity in comprehending the mechanisms by which these minute plastic particles might carry HMs and influence their dispersion and accessibility. It is crucial to enhance several techniques that aid in reducing and managing the stress caused by harmful substances in plants. Using a single technique is ultimately unrealistic and inadequate for effectively restoring soil that has been polluted with HMs (Priya et al., 2023). There have been numerous methods developed to mitigate or prevent HM pollution and to reestablish vegetation in polluted soil (Priya et al., 2023).

Restoring the flora of soil that has been contaminated with HMs is a highly promising approach known as phytoremediation (Yan et al., 2020). Public acceptance has been achieved, and it offers numerous advantages over other physicochemical treatments (Mitra et al., 2022).

The most effective and cost-efficient approach is the recent emergence of the introduction of nanoparticles (NPs) into plants to increase their tolerance to HM toxicity and facilitate the cleansing of these toxic elements (Zhou et al., 2020). Genetic engineering is a valuable method for modifying plants to manifest specific characteristics, including rapid growth, high biomass output, strong tolerance and accumulation of HMs, and adaptability to a variety of climatic and geological conditions (Yan et al., 2020). As a result, it will be essential to have a thorough understanding of the processes by which plants absorb, transport, and eliminate HMs as well as the identification and analysis of a variety of molecules and signaling pathways in order to create genetically engineered plant species that are optimal for phytoremediation (Priya et al., 2023). Enhanced tolerance or accumulation of HMs in plants may be achieved by manipulating genes associated with HM absorption, translocation, sequestration, and tolerance. In addition, the bioavailability of HMs may be improved by the use of chelating compounds and microorganisms, which, in turn, facilitates their accumulation in plants (Olaniran et al., 2013). In addition, they can be employed to improve the health of the soil and to further encourage the growth and fitness of plants. Several hyperaccumulator plants have been identified, and the most direct method for phytoremediation is the use of HM hyperaccumulators (Skuza et al., 2022). However, there are certain limitations that impede the utilization of these natural hyperaccumulators in phytoremediation (Yan et al., 2020).

Due to their capacity to penetrate plants extensively, exhibit superior adsorption, and deliver targeted effects, they may be instrumental in the regulation of photosynthesis and the detoxification of ROS (Rasheed et al., 2022). Subsequently, they can substantially improve the germination, growth, and yield of plant seeds (Kornarzyński et al., 2020). NPs also facilitate plant growth by modulating the movement and distribution of both mobile and immobile forms of HMs (Zhou et al., 2020). The potential of NPs to significantly improve the remediation of metal-contaminated soils in the future is suggested by the positive results observed in the use of NPs, particularly in the enhancement of plants’ resistance to HMs and the facilitation of their development.

## 9 Conclusion

HMs are defined as metals with densities more than 5 g cm^3^. HMs account for 53 of the almost 90 elements found in nature. Plant nutrition is thought to need a grand total of 18 components. Some of its parts are thought to have positive effects. HMs in the soil can have a negative impact on human and animal health, soil quality, fertility, and agricultural productivity. Plants have developed ways to deal with HM stress, such as immobilization, exclusion outside the plasma membrane, limited absorption and transport, production of specific HM transporters, activation of stress proteins, and chelation and sequestration by specific ligands.

Metals have two distinct impacts on seed germination: their overall toxicity and their ability to hinder water uptake. Plants absorb and store HMs using concentration gradients and selective absorption. These chemicals influence enzymes, cellular metabolism, and the production of nucleic acids, proteins, and pigments for photosynthesis. Recent molecular and genetic research has identified gene families that play a role in metal transport, contributing to metal tolerance in hyperaccumulator plants. Understanding the interactions between different HMs in plants is crucial for assessing and controlling risks.

Research on agricultural genetic diversity and plant adaptation mechanisms is also essential for developing metal-tolerant agricultural cultivars and reducing the harmful impact of contaminated soil on crop growth and productivity. Finally, the current review examined the locations in plants where HMs are found. It discussed the benefits of HMs to plants, the consequences on seed performance, and the initial plant reaction to HM exposure. This review also looks at the existence of HM transport genes in plants and how plants react to HMs on a molecular, biochemical, and metabolic level, respectively. Methods for managing HMs in plants, together with the associated challenges and opportunities, have been also examined.

## References

[B1] Abbas M. Li Y. Elbaiomy R. G. Yan K. Ragauskas A. J. Yadav V.. (2022). Genome-wide analysis and expression profiling of SlHsp70 gene family in *Solanum lycopersicum* revealed higher expression of SlHsp70-11 in roots under Cd^2+^ stress. Front. Biosci. 27, 186. doi: 10.31083/j.fbl2706186 35748262

[B2] Abd Elnabi M. K. Elkaliny N. E. Elyazied M. M. Azab S. H. Elkhalifa S. A. Elmasry S.. (2023). Toxicity of heavy metals and recent advances in their removal: a review. Toxics 11, 580. doi: 10.3390/toxics11070580 37505546 PMC10384455

[B3] Adnan M. Xiao B. Ali M. U. Xiao P. Zhao P. Wang H.. (2024). Heavy metals pollution from smelting activities: a threat to soil and groundwater. Ecotoxicol. Environ. Saf. 274, 116189. doi: 10.1016/j.ecoenv.2024.116189 38461579

[B4] Adrees M. Ali S. Rizwan M. Ibrahim M. Abbas F. Farid M.. (2015). The effect of excess copper on growth and physiology of important food crops: a review. Environ. Sci. pollut. Res. 22, 8148–8162doi: 10.1007/s11356-015-4496-5 25874438

[B5] Alam M. M. Hayat S. Ali B. Ahmad A. (2007). Effect of 28-homobrassinolide treatment on nickel toxicity in *Brassica juncea* . Photosynthetica 45, 139–142. doi: 10.1007/s11099-007-0022-4

[B6] Alejandro S. Höller S. Meier B. Peiter E. (2020). Manganese in plants: from acquisition to subcellular allocation. Front. Plant Sci. 11, 300. doi: 10.3389/fpls.2020.00300 32273877 PMC7113377

[B7] Alengebawy A. Abdelkhalek S. T. Qureshi S. R. Wang M. Q. (2021). Heavy metals and pesticides toxicity in agricultural soil and plants: ecological risks and human health implications. Toxics 9, 42. doi: 10.3390/toxics9030042 33668829 PMC7996329

[B8] Ali H. Khan E. (2018). What are heavy metals? Long-standing controversy over the scientific use of the term ‘heavy metals’ – proposal of a comprehensive definition. Toxicol. Environ. Chem. 100, 6–19. doi: 10.1080/02772248.2017.1413652

[B9] Angon P. B. Islam M. S. Kc S. Das A. Anjum N. Poudel A. (2024). Sources, effects and present perspectives of heavy metals contamination: soil, plants and human food chain. Heliyon 10, e28357. doi: 10.1016/j.heliyon.2024.e28357 38590838 PMC10999863

[B10] Angulo-Bejarano P. I. Puente-Rivera J. Cruz-Ortega R. (2021). Metal and metalloid toxicity in plants: an overview on molecular aspects. Plants 10, 635. doi: 10.3390/plants10040635 33801570 PMC8066251

[B11] Aponte H. Meli P. Butler B. Paolini J. Matus F. Merino C.. (2020). Meta-analysis of heavy metal effects on soil enzyme activities. Sci. Total Environ. 737, 139744. doi: 10.1016/j.scitotenv.2020.139744 32512304

[B12] Arif N. Yadav V. Singh S. Singh S. Ahmad P. Mishra R. K.. (2016). Influence of high and low levels of plant-beneficial heavy metal ions on plant growth and development. Front. Environ. Sci. 4, 69. doi: 10.3389/fenvs.2016.00069

[B13] Arrivault S. Senger T. Krämer U. (2006). The *Arabidopsis* metal tolerance protein AtMTP3 maintains metal homeostasis by mediating Zn exclusion from the shoot under Fe deficiency and Zn oversupply. Plant J. 46, 861–879. doi: 10.1111/j.1365-313X.2006.02746.x 16709200

[B14] Asiminicesei D. M. Fertu D. I. Gavrilescu M. (2024). Impact of heavy metal pollution in the environment on the metabolic profile of medicinal plants and their therapeutic potential. Plants 13, 913. doi: 10.3390/plants13060913 38592933 PMC10976221

[B15] Baker A. J. M. (1981). Accumulators and excluders strategies in response of plants to heavy metals. J. Plant Nutr. 3, 643–654. doi: 10.1080/01904168109362867

[B16] Batool T. S. Aslam R. Gul A. Paracha R. Z. Ilyas M. De Abreu K.. (2023). Genome-wide analysis of heavy metal ATPases (HMAs) in Poaceae species and their potential role against copper stress in *Triticum aestivum* . Sci. Rep. 13, 7551. doi: 10.1038/s41598-023-32023-7 37160901 PMC10170112

[B17] Begum W. Rai S. Banerjee S. Bhattacharjee S. Mondal M. H. Bhattarai A.. (2022). A comprehensive review on the sources, essentiality and toxicological profile of nickel. RSC. Adv. 12, 9139–9153. doi: 10.1039/D2RA00378C 35424851 PMC8985085

[B18] Bhati K. K. Sharma S. Aggarwal S. Kaur M. Shukla V. Kaur J.. (2015). Genome-wide identification and expression characterization of ABCC-MRP transporters in hexaploid wheat. Front. Plant Sci. 6, 488. doi: 10.3389/fpls.2015.00488 26191068 PMC4486771

[B19] Bhattacharjee B. Ali A. Tuteja N. Gill S. Pattanayak A. (2023). Identification and expression pattern of aluminum-responsive genes in roots of rice genotype with reference to Al-sensitivity. Sci. Rep. 13, 12184. doi: 10.1038/s41598-023-39238-8 37500702 PMC10374657

[B20] Bhattacharyya A. Chattopadhyay R. Mitra S. Crowe S. E. (2014). Oxidative stress: an essential factor in the pathogenesis of gastrointestinal mucosal diseases. Physiol. Rev. 94, 329–354. doi: 10.1152/physrev.00040.2012 24692350 PMC4044300

[B21] Bielen A. Remans T. Vangronsveld J. Cuypers A. (2013). The influence of metal stress on the availability and redox state of ascorbate, and possible interference with its cellular functions. Int. J. Mol. Sci. 14, 6382–6413. doi: 10.3390/ijms14036382 23519107 PMC3634492

[B22] Bortoloti G. A. Baron D. (2022). Phytoremediation of toxic heavy metals by *Brassica* plants: a biochemical and physiological approach. Environ. Adv. 8, 100204. doi: 10.1016/j.envadv.2022.100204

[B23] Branca J. J. V. Fiorillo C. Carrino D. Paternostro F. Taddei N. Gulisano M.. (2020). Cadmium-induced oxidative stress: focus on the central nervous system. Antioxidants 9, 492. doi: 10.3390/antiox9060492 32516892 PMC7346204

[B24] Bressler J. P. Olivi L. Cheong J. H. Kim Y. Maerten A. Bannon D. (2007). Metal transporters in intestine and brain: their involvement in metal-associated neurotoxicities. Hum. Exp. Toxicol. 26, 221–229. doi: 10.1177/0960327107070573 17439925

[B25] Brookes A. Collins J. C. Thurman D. A. (1981). The mechanism of zinc tolerance in grasses. *J* . Plant Nutr. 3, 695–705. doi: 10.1080/01904168109362872

[B26] Brunetti P. Zanella L. De Paolis A. Di Litta D. Cecchetti V. Falasca G.. (2015). Cadmium-inducible expression of the ABC-type transporter AtABCC3 increases phytochelatin-mediated cadmium tolerance in *Arabidopsis* . J. Exp. Bot. 66, 3815–3829. doi: 10.1093/jxb/erv185 25900618 PMC4473984

[B27] Bursakov S. A. Kroupin P. Y. Karlov G. I. Divashuk M. G. (2023). Tracing the element: the molecular bases of molybdenum homeostasis in legumes. Agronomy 13, 2300. doi: 10.3390/agronomy13092300

[B28] Calderón-Vázquez C. Sawers R. J. Herrera-Estrella L. (2011). Phosphate deprivation in maize: genetics and genomics. Plant Physiol. 156, 1067–1077. doi: 10.1104/pp.111.174987 21617030 PMC3135936

[B29] Castrillo G. Sánchez-Bermejo E. de Lorenzo L. Crevillén P. Fraile-Escanciano A. Tc M.. (2013). WRKY6 transcription factor restricts arsenate uptake and transposon activation in *Arabidopsis* . Plant Cell 25, 2944–2957. doi: 10.1105/tpc.113.114009 23922208 PMC3784590

[B30] Chen G. Li J. Han H. Du R. Wang X. (2022). Physiological and molecular mechanisms of plant responses to copper stress. Int. J. Mol. Sci. 23, 12950. doi: 10.3390/ijms232112950 36361744 PMC9656524

[B31] Chen J. Wang J. W. Shu Y. H. (2020a). Review on the effects of heavy metal pollution on herbivorous insects. Ying Yong Sheng Tai Xue Bao 31, 1773–1782. doi: 10.13287/j.1001-9332.202005.035 32530257

[B32] Chao L. M. Liu Y. Q. Chen D. Y. Xue X. Y. Mao Y. B. Chen X. Y. (2017). *Arabidopsis* transcription factors SPL1 and SPL12 confer plant thermotolerance at reproductive stage. Mol. Plant 10, 735–748. doi: 10.1016/j.molp.2017.03.010 28400323

[B33] Chen P. Chen T. Li Z. Jia R. Luo D. Tang M.. (2020b). Transcriptome analysis revealed key genes and pathways related to cadmium-stress tolerance in Kenaf (*Hibiscus cannabinus* L.). Ind. Crop Prod. 158, 112970. doi: 10.1016/j.indcrop.2020.112970

[B34] Chen Y. Wang Y. Wu W. Lin Q. Xue S. (2006). Impacts of chelate-assisted phytoremediation on microbial community composition in the rhizosphere of a copper accumulator and non-accumulator. Sci. Total Environ. 356, 247–255. doi: 10.1016/j.scitotenv.2005.04.028 15935447

[B35] Chen Y. T. Wang Y. Yeh K. C. (2017). Role of root exudates in metal acquisition and tolerance. Curr. Opin. Plant Biol. 39, 66–72. doi: 10.1016/j.pbi.2017.06.004 28654805

[B36] Chen J. Yang L. Gu J. Bai X. Ren Y. Fan T.. (2015). MAN 3 gene regulates cadmium tolerance through the glutathione-dependent pathway in *Arabidopsis thaliana* . New Phytol. 205, 570–582. doi: 10.1111/nph.13101 25329733

[B37] Chiang H. C. Lo J. C. Yeh K. C. (2006). Genes associated with heavy metal tolerance and accumulation in Zn/Cd hyperaccumulator *Arabidopsis halleri*: a genomic survey with cDNA microarray. Environ. Sci. Technol. 40, 6792–6798. doi: 10.1021/es061432y 17144312

[B38] Claus J. Bohmann A. Chavarría-Krauser A. (2013). Zinc uptake and radial transport in roots of *Arabidopsis thaliana*: a modelling approach to understand accumulation. Ann. Bot. 112, 369–380. doi: 10.1093/aob/mcs263 23258417 PMC3698380

[B39] Clemens S. (2001). Molecular mechanisms of plant metal tolerance and homeostasis. Planta 212, 475–486. doi: 10.1007/s004250000458 11525504

[B40] Cohen C. K. Norvell W. A. Kochian L. V. (1997). Induction of the root cell plasma membrane ferric reductase (an exclusive role for Fe and Cu). Plant Physiol. 114, 1061–1069. doi: 10.1104/pp.114.3.1061 12223760 PMC158395

[B41] Collin F. (2019). Chemical basis of reactive oxygen species reactivity and involvement in neurodegenerative diseases. Int. J. Mol. Sci. 20 (10), 2407. doi: 10.3390/ijms20102407 31096608 PMC6566277

[B42] Collin S. Baskar A. Geevarghese D. M. Ali M. N. V. S. Bahubali P. Choudhary R.. (2022). Bioaccumulation of lead (Pb) and its effects in plants: a review. J. Hazard. Mater. Lett. 3, 100064. doi: 10.1016/j.hazl.2022.100064

[B43] Costa M. I. Sarmento-Ribeiro A. B. Gonçalves A. C. (2023). Zinc: from biological functions to therapeutic potential. Int. J. Mol. Sci. 24 (5), 4822. doi: 10.3390/ijms24054822 36902254 PMC10003636

[B44] Dai J. Becquer T. Rouiller J. H. Reversat G. Bernhard-Reversat F. Lavelle P. (2004). Influence of heavy metals on C and N mineralization and microbial biomass in Zn-, Pb-, Cu-, and Cd-contaminated soils. Appl. Soil Ecol. 25, 99–109. doi: 10.1016/j.apsoil.2003.09.003

[B45] DalCorso G. Farinati S. Maistri S. Furini A. (2008). How plants cope with cadmium: staking all on metabolism and gene expression. J. Integr. Plant Biol. 50, 1268–1280. doi: 10.1111/j.1744-7909.2008.00737.x 19017114

[B46] Danouche M. El Ghachtouli N. El Arroussi H. (2021). Phycoremediation mechanisms of heavy metals using living green microalgae: physicochemical and molecular approaches for enhancing selectivity and removal capacity. Heliyon 7, e07609. doi: 10.1016/j.heliyon.2021.e07609 34355100 PMC8322293

[B47] Daspute A. A. Sadhukhan A. Tokizawa M. Kobayashi Y. Panda S. K. Koyama H. (2017). Transcriptional regulation of aluminum-tolerance genes in higher plants: clarifying the underlying molecular mechanisms. Front. Plant Sci. 8, 1358. doi: 10.3389/fpls.2017.01358 28848571 PMC5550694

[B48] De Caroli M. Furini A. DalCorso G. Rojas M. Di Sansebastiano G. P. (2020). Endomembrane reorganization induced by heavy metals. Plants 9, 482. doi: 10.3390/plants9040482 32283794 PMC7238196

[B49] Delhaize E. Gruber B. D. Pittman J. K. White R. G. Leung H. Miao Y.. (2007). A role for the AtMTP11 gene of *Arabidopsis* in manganese transport and tolerance. Plant J. 51, 198–210. doi: 10.1111/j.1365-313X.2007.03138.x 17559518

[B50] Desbrosses-Fonrouge A. G. Voigt K. Schröder A. Arrivault S. Thomine S. Krämer U. (2005). *Arabidopsis thaliana* MTP1 is a Zn transporter in the vacuolar membrane which mediates Zn detoxification and drives leaf Zn accumulation. FEBS. Lett. 579, 4165–4174. doi: 10.1016/j.febslet.2005.06.046 16038907

[B51] Dixit R. Wasiullah X. Malaviya D. Pandiyan K. Singh U. B. Sahu A.. (2015). Bioremediation of heavy metals from soil and aquatic environment: an overview of principles and criteria of fundamental processes. Sustainability 7, 2189–2212. doi: 10.3390/su7022189

[B52] Dräger D. B. Desbrosses-Fonrouge A. G. Krach C. Chardonnens A. N. Meyer R. C. Saumitou-Laprade P.. (2004). Two genes encoding *Arabidopsis halleri* MTP1 metal transport proteins co-segregate with zinc tolerance and account for high MTP1 transcript levels. Plant J. 39, 425–439. doi: 10.1111/j.1365-313X.2004.02143.x 15255871

[B53] Drew D. North R. A. Nagarathinam K. Tanabe M. (2021). Structures and general transport mechanisms by the major facilitator superfamily (MFS). *Chem* . Rev. 121, 5289–5335. doi: 10.1021/acs.chemrev.0c00983 PMC8154325 33886296

[B54] Du Z. Y. Chen M. X. Chen Q. F. Gu J. D. Chye M. L. (2015). Expression of *Arabidopsis* acyl-CoA-binding proteins AtACBP 1 and AtACBP 4 confers P b (II) accumulation in *Brassica juncea* roots. Plant Cell Environ. 38, 101–117. doi: 10.1111/pce.12382 24906022

[B55] Dumanović J. Nepovimova E. Natić M. Kuča K. Jaćević V. (2020). The significance of reactive oxygen species and antioxidant defense system in plants: a concise overview. Front Plant Sci. 11, 552969. doi: 10.3389/fpls.2020.552969 33488637 PMC7815643

[B56] Dutta S. Mitra M. Agarwal P. Mahapatra K. De S. Sett U.. (2018). Oxidative and genotoxic damages in plants in response to heavy metal stress and maintenance of genome stability. Plant Signal. Behav. 13, e1460048. doi: 10.1080/15592324.2018.1460048 29621424 PMC6149466

[B57] El-Sappah A. H. Abbas M. Rather S. A. Wani S. H. Soaud N. Noor Z.. (2023). Genome-wide identification and expression analysis of metal tolerance protein (MTP) gene family in soybean (*Glycine max*) under heavy metal stress. Mol. Biol. Rep. 502975, 2990doi: 10.1007/s11033-022-08100-x 36653731

[B58] El-Sappah A. H. Elbaiomy R. G. Elrys A. S. Wang Y. Zhu Y. Huang Q.. (2021a). Genome-wide identification and expression analysis of metal tolerance protein gene family in *Medicago truncatula* under a broad range of heavy metal stress. Front. Genet. 12, 713224. doi: 10.3389/fgene.2021.713224 34603378 PMC8482800

[B59] El-Sappah A. H. Elrys A. S. Desoky E. M. Zhao X. Bingwen W. El-Sappah H. H.. (2021b). Comprehensive genome wide identification and expression analysis of MTP gene family in tomato (*Solanum lycopersicum*) under multiple heavy metal stress. Saudi J. Biol. Sci. 28, 6946–6956. doi: 10.1016/j.sjbs.2021.07.073 34866994 PMC8626246

[B60] El-Sappah A. H. Rather S. A. (2022). “Genomics approaches to study abiotic stress tolerance in plants,” in Plant abiotic stress physiology. Eds. Aftab T. Hakeem K. R. (CRC Press, Boca Raton, FL, USA), 25–46. doi: 10.1201/9781003180579

[B61] El-Sappah A. H. Shawky A. S. H. Sayed-Ahmad M. S. Youssef M. A. H. (2017). Estimation of heat shock protein 70 (hsp 70) gene expression in Nile tilapia (*Oreochromis niloticus*) using quantitative Real-Time PCR. *Zagazig J.Agric* . Res. 44, 1003–1015. doi: 10.21608/zjar.2017.52300

[B62] Emamverdian A. Ding Y. Mokhberdoran F. Xie Y. (2015). Heavy metal stress and some mechanisms of plant defense response. Sci. World J. 2015, 756120. doi: 10.1155/2015/756120 PMC4321847 25688377

[B63] Ercal N. Gurer-Orhan H. Aykin-Burns N. (2001). Toxic metals and oxidative stress part I: mechanisms involved in metal-induced oxidative damage. Curr. Top. Med. Chem. 1, 529–539. doi: 10.2174/1568026013394831 11895129

[B64] Ezaki B. Gardner R. C. Ezaki Y. Matsumoto H. (2000). Expression of aluminum-induced genes in transgenic *Arabidopsis* plants can ameliorate aluminum stress and/or oxidative stress. Plant Physiol. 122, 657–666. doi: 10.1104/pp.122.3.657 10712528 PMC58900

[B65] Faizan M. Alam P. Hussain A. Karabulut F. Tonny S. H. Cheng S. H.. (2024). Phytochelatins: key regulator against heavy metal toxicity in plants. Plant Stress 11, 100355. doi: 10.1016/j.stress.2024.100355

[B66] Fan X. Zhou X. Chen H. Tang M. Xie X. (2021). Cross-talks between macro- and micronutrient uptake and signaling in plants. Front. Plant Sci. 12, 663477. doi: 10.3389/fpls.2021.663477 34721446 PMC8555580

[B67] Feng Ma J. Hiradate S. Matsumoto H. (1998). High aluminum resistance in buckwheat. Ii. Oxalic acid detoxifies aluminum internally. Plant Physiol. 117, 753–759. doi: 10.1104/pp.117.3.753 9662518 PMC34930

[B68] Festa R. A. Thiele D. J. (2011). Copper: an essential metal in biology. Curr. Biol. 21, R877–R883. doi: 10.1016/j.cub.2011.09.040 22075424 PMC3718004

[B69] Foyer C. H. Rasool B. Davey J. W. Hancock R. D. (2016). Cross-tolerance to biotic and abiotic stresses in plants: a focus on resistance to aphid infestation. J. Exp. Bot. 67, 2025–2037. doi: 10.1093/jxb/erw079 26936830

[B70] Freisinger E. (2011). Structural features specific to plant metallothioneins. J. Biol. Inorg. Chem. 16, 1035–1045. doi: 10.1007/s00775-011-0801-z 21688177

[B71] Gallo-Franco J. J. Sosa C. C. Ghneim-Herrera T. Quimbaya M. (2020). Epigenetic control of plant response to heavy metal stress: a new view on aluminum tolerance. Front. Plant Sci. 11, 602625. doi: 10.3389/fpls.2020.602625 33391313 PMC7772216

[B72] García A. Baquedano F. J. Navarro P. Castillo F. J. (1999). Oxidative stress induced by copper in sunflower plants. Free Radic. Res. 31, S45–S50. doi: 10.1080/10715769900301311 10694040

[B73] Garcia-Molina A. Andrés-Colás N. Perea-García A. Del Valle-Tascón S. Peñarrubia L. Puig S. (2011). The intracellular *Arabidopsis* COPT5 transport protein is required for photosynthetic electron transport under severe copper deficiency. Plant J. 65, 848–860. doi: 10.1111/j.1365-313X.2010.04472.x 21281364

[B74] Garcia-Molina A. Andrés-Colás N. Perea-García A. Neumann U. Dodani S. C. Huijser P.. (2013). The *Arabidopsis* COPT6 transport protein functions in copper distribution under copper-deficient conditions. Plant Cell Physiol. 54, 1378–1390. doi: 10.1093/pcp/pct088 23766354

[B75] Gasic K. Korban S. S. (2006). “Heavy metal stress,” in Physiology and molecular biology of stress tolerance in plants, vol. pp . Eds. Rao M. Raghavendra K. Janardhan A. Reddy K. (Springer, Dordrecht. Netherlands), 219–254. doi: 10.1007/1-4020-4225-6_8219-254

[B76] Genchi G. Sinicropi M. S. Lauria G. Carocci A. Catalano A. (2020). The effects of cadmium toxicity. Int. J. Environ. Res. Public Health 17 (11), 3782. doi: 10.3390/ijerph17113782 32466586 PMC7312803

[B77] Gill R. A. Ahmar S. Ali B. Saleem M. H. Khan M. U. Zhou W.. (2021). The role of membrane transporters in plant growth and development, and abiotic stress tolerance. Int. J. Mol. Sci. 22, 12792. doi: 10.3390/ijms222312792 34884597 PMC8657488

[B78] Gill S. S. Khan N. A. Tuteja N. (2012). Cadmium at high dose perturbs growth, photosynthesis and nitrogen metabolism while at low dose it up regulates sulfur assimilation and antioxidant machinery in garden cress (*Lepidium sativum* L.). Plant Sci. 182, 112–120. doi: 10.1016/j.plantsci.2011.04.018 22118622

[B79] Giri A. K. Patel R. K. (2011). Toxicity and bioaccumulation potential of Cr (VI) and Hg (II) on differential concentration by *Eichhornia crassipes* in hydroponic culture. Water Sci. Technol. 63, 899–907. doi: 10.2166/wst.2011.268 21411939

[B80] Goyer R. Golub M. Choudhury H. Hughes M. Kenyon E. Stifelman M. (2004). “Issue paper on the human health effects of metals,” in US environmental protection agency risk assessment forum (ERG, Lexington, KY, USA), 1200.

[B81] Grotz N. Fox T. Connolly E. Park W. Guerinot M. L. Eide D. (1998). Identification of a family of zinc transporter genes from *Arabidopsis* that respond to zinc deficiency. Proc. Natl. Acad. Sci. U.S.A. 95, 7220–7224. doi: 10.1073/pnas.95.12.7220 9618566 PMC22785

[B82] Guerinot M. L. (2000). The ZIP family of metal transporters. Biochim. Biophys. Acta 1465, 190–198. doi: 10.1016/S0005-2736(00)00138-3 10748254

[B83] Gulcin İ. Alwasel S. (2022). Metal ions, metal chelators and metal chelating assay as antioxidant method. Processes 10, 132. doi: 10.3390/pr10010132

[B84] Guo W. J. Bundithya W. Goldsbrough P. B. (2003). Characterization of the *Arabidopsis* metallothionein gene family: tissue-specific expression and induction during senescence and in response to copper. New Phytol. 159, 369–381. doi: 10.1046/j.1469-8137.2003.00813.x 33873353

[B85] Hajam Y. A. Kumar R. Kumar A. (2023). Environmental waste management strategies and vermi transformation for sustainable development. Environ. Challen. 13, 100747. doi: 10.1016/j.envc.2023.100747

[B86] Hall J. L. (2002). Cellular mechanisms for heavy metal detoxification and tolerance. J. Exp. Bot. 53, 1–11. doi: 10.1093/jexbot/53.366.1 11741035

[B87] Hama Aziz K. H. Mustafa F. S. Omer K. M. Hama S. Hamarawf R. F. Rahman K. O. (2023). Heavy metal pollution in the aquatic environment: efficient and low-cost removal approaches to eliminate their toxicity: a review. RSC. Adv. 13, 17595–17610. doi: 10.1039/D3RA00723E 37312989 PMC10258679

[B88] Hamsa N. Yogesh G. S. Koushik U. Patil L. (2017). Nitrogen transformation in soil: effect of heavy metals. Int. J. Curr. Microbiol. Appl. Sci. 6, 816–832. doi: 10.20546/ijcmas.2017.605.092

[B89] Hamzah Saleem M. Usman K. Rizwan M. Al Jabri H. Alsafran M. (2022). Functions and strategies for enhancing zinc availability in plants for sustainable agriculture. Front. Plant Sci. 13, 1033092. doi: 10.3389/fpls.2022.1033092 36275511 PMC9586378

[B90] Hasan M. K. Cheng Y. Kanwar M. K. Chu X. Y. Ahammed G. J. Qi Z. Y. (2017). Responses of plant proteins to heavy metal stress-a review. Front.Plant Sci. 8, 1492. doi: 10.3389/fpls.2017.01492 28928754 PMC5591867

[B91] Hasanuzzaman M. Alhaithloul H. A. S. Parvin K. Bhuyan M. B. Tanveer M. Mohsin S. M.. (2019). Polyamine action under metal/metalloid stress: regulation of biosynthesis, metabolism, and molecular interactions. Int. J. Mol. Sci. 20 (13), 3215. doi: 10.3390/ijms20133215 31261998 PMC6651247

[B92] Hasanuzzaman M. Bhuyan M. B. Zulfiqar F. Raza A. Mohsin S. M. Mahmud J. A.. (2020). Reactive oxygen species and antioxidant defense in plants under abiotic stress: revisiting the crucial role of a universal defense regulator. Antioxidants 9, 681. doi: 10.3390/antiox9080681 32751256 PMC7465626

[B93] Hasanuzzaman M. Nahar K. Alam M. M. Roychowdhury R. Fujita M. (2013). Physiological, biochemical, and molecular mechanisms of heat stress tolerance in plants. Int. J. Mol. Sci. 14, 9643–9684. doi: 10.3390/ijms14059643 23644891 PMC3676804

[B94] Hashimoto H. Uragami C. Cogdell R. J. (2016). Carotenoids and photosynthesis. Subcell. Biochem. 79, 111–139. doi: 10.1007/978-3-319-39126-7_4 27485220

[B95] Hayat S. Hayat Q. AlYemeni M. N. Wani A. S. Pichtel J. Ahmad A. (2012). Role of proline under changing environments: a review. Plant Signal. Behav. 7, 1456–1466. doi: 10.4161/psb.21949 22951402 PMC3548871

[B96] Haydon M. J. Cobbett C. S. (2007). A novel major facilitator superfamily protein at the tonoplast influences zinc tolerance and accumulation in *Arabidopsis* . Plant Physiol. 143, 1705–1719. doi: 10.1104/pp.106.092015 17277087 PMC1851824

[B97] Hinsinger P. Plassard C. Tang C. Jaillard B. (2003). Origins of root-mediated pH changes in the rhizosphere and their responses to environmental constraints: a review. Plant Soil. 248, 43–59. doi: 10.1023/A:1022371130939

[B98] Hossain M. A. Piyatida P. da Silva J. A. T. Fujita M. (2012). Molecular mechanism of heavy metal toxicity and tolerance in plants: central role of glutathione in detoxification of reactive oxygen species and methylglyoxal and in heavy metal chelation. J. Bot. 2012, 872875. doi: 10.1155/2012/872875

[B99] Hu S. Yu Y. Chen Q. Mu G. Shen Z. Zheng L. (2017). OsMYB45 plays an important role in rice resistance to cadmium stress. Plant Sci. 264, 1–8. doi: 10.1016/j.plantsci.2017.08.002 28969789

[B100] Huang Y. Adeleye A. S. Zhao L. Minakova A. S. Anumol T. Keller A. A. (2019). Antioxidant response of cucumber (*Cucumis sativus*) exposed to nano copper pesticide: quantitative determination via LC-MS/MS. Food Chem. 270, 47–52. doi: 10.1016/j.foodchem.2018.07.069 30174074

[B101] Huang D. Huo J. Liao W. (2021). Hydrogen sulfide: roles in plant abiotic stress response and crosstalk with other signals. Plant Sci. 302, 110733. doi: 10.1016/j.plantsci.2020.110733 33288031

[B102] Igiri B. E. Okoduwa S. I. R. Idoko G. O. Akabuogu E. P. Adeyi A. O. Ejiogu I. K. (2018). Toxicity and bioremediation of heavy metals contaminated ecosystem from tannery wastewater: a review. J. Toxicol. 2018, 2568038. doi: 10.1155/2018/2568038 30363677 PMC6180975

[B103] Islam M. A. Guo J. Peng H. Tian S. Bai X. Zhu H.. (2020). TaYS1A, a yellow stripe-like transporter gene, is required for wheat resistance to *Puccinia striiformis* f.sp. Tritici. Genes 11 (12), 1452. doi: 10.3390/genes11121452 33287151 PMC7761651

[B104] Jadia C. D. Fulekar M. H. (2009). Phytoremediation of heavy metals: recent techniques. Afr. J. Biotechnol. 8, 921–928.

[B105] Jaishankar M. Tseten T. Anbalagan N. Mathew B. B. Beeregowda K. N. (2014). Toxicity, mechanism and health effects of some heavy metals. *Interdiscip* . Toxicol. 7, 60–72. doi: 10.2478/intox-2014-0009 PMC4427717 26109881

[B106] Jiang X. Li M. (2020). “Chapter 5 - Ecological safety hazards of wastewater,” in High-risk pollutants in wastewater. Eds. Ren H. Zhang. X. (Elsevier, Amsterdam, Netherlands), 101–123.

[B107] Jogawat A. Chhaya Y. B. Narayan O. P. (2021). Metal transporters in organelles and their roles in heavy metal transportation and sequestration mechanisms in plants. Physiol. Plant 173, 259–275. doi: 10.1111/ppl.13370 33586164

[B108] Jomova K. Raptova R. Alomar S. Y. Alwasel S. H. Nepovimova E. Kuca K.. (2023). Reactive oxygen species, toxicity, oxidative stress, and antioxidants: chronic diseases and aging. Arch. Toxicol. 97, 2499–2574. doi: 10.1007/s00204-023-03562-9 37597078 PMC10475008

[B109] Kaczmarek M. Cachau R. E. Topol I. A. Kasprzak K. S. Ghio A. Salnikow K. (2009). Metal ions-stimulated iron oxidation in hydroxylases facilitates stabilization of HIF-1 alpha protein. Toxicol. Sci. 107, 394–403. doi: 10.1093/toxsci/kfn251 19074761 PMC2639755

[B110] Kafle A. Timilsina A. Gautam A. Adhikari K. Bhattarai A. Aryal N. (2022). Phytoremediation: mechanisms, plant selection and enhancement by natural and synthetic agents. Environ. Adv. 8, 100203. doi: 10.1016/j.envadv.2022.100203

[B111] Kajla M. Roy A. Singh I. K. Singh A. (2023). Regulation of the regulators: transcription factors controlling biosynthesis of plant secondary metabolites during biotic stresses and their regulation by miRNAs. Front. Plant Sci. 14, 1126567. doi: 10.3389/fpls.2023.1126567 36938003 PMC10017880

[B112] Khan F. Siddique A. B. Shabala S. Zhou M. Zhao C. (2023). Phosphorus plays key roles in regulating plants’ physiological responses to abiotic stresses. Plants 12 (15), 2861. doi: 10.3390/plants12152861 37571014 PMC10421280

[B113] Klatte M. Schuler M. Wirtz M. Fink-Straube C. Hell R. Bauer P. (2009). The analysis of *Arabidopsis nicotianamine* synthase mutants reveals functions for nicotianamine in seed iron loading and iron deficiency responses. Plant Physiol. 150, 257–271. doi: 10.1104/pp.109.136374 19304929 PMC2675739

[B114] Kobae Y. Uemura T. Sato M. H. Ohnishi M. Mimura T. Nakagawa T.. (2004). Zinc transporter of *Arabidopsis thaliana* AtMTP1 is localized to vacuolar membranes and implicated in zinc homeostasis. Plant Cell Physiol. 45, 1749–1758. doi: 10.1093/pcp/pci015 15653794

[B115] Kolaj-Robin O. Russell D. Hayes K. A. Pembroke J. T. Soulimane T. (2015). Cation diffusion facilitator family: structure and function. FEBS. Letters. 589, 1283–1295. doi: 10.1016/j.febslet.2015.04.007 25896018

[B116] Koren’Kov V. Park S. Cheng N. H. Sreevidya C. Lachmansingh J. Morris J.. (2007). Enhanced Cd^2+^-selective root-tonoplast-transport in tobaccos expressing *Arabidopsis* cation exchangers. Planta 225, 403–411. doi: 10.1007/s00425-006-0352-7 16845524

[B117] Kornarzyński K. Sujak A. Czernel G. Wiącek D. (2020). Effect of Fe_3_O_4_ nanoparticles on germination of seeds and concentration of elements in *Helianthus annuus* L. under constant magnetic field. Sci. Rep. 10, 8068. doi: 10.1038/s41598-020-64849-w 32415165 PMC7228974

[B118] Kostenkova K. Scalese G. Gambino D. Crans D. C. (2022). Highlighting the roles of transition metals and speciation in chemical biology. Curr. Opin. Chem. Biol. 69, 102155. doi: 10.1016/j.cbpa.2022.102155 35643024

[B119] Kraemer U. (2009). The dilemma of controlling heavy metal accumulation in plants. New Phytol. 181, 3–5. doi: 10.1111/j.1469-8137.2008.02699.x 19076712

[B120] Kranner I. Colville L. (2011). Metals and seeds: biochemical and molecular implications and their significance for seed germination. Environ. Exp. Bot. 72, 93–105. doi: 10.1016/j.envexpbot.2010.05.005

[B121] Kudo H. Qian Z. Inoue C. Chien M. F. (2023). Temperature dependence of metals accumulation and removal kinetics by *Arabidopsis halleri* ssp. *gemmifera* . Plants 12, 877. doi: 10.3390/plants12040877 36840224 PMC9966424

[B122] Kumar V. Roy S. Behera B. K. Das B. K. (2022). Heat shock proteins (Hsps) in cellular homeostasis: a promising tool for health management in crustacean aquaculture. Life 12 (11), 1777. doi: 10.3390/life12111777 36362932 PMC9699388

[B123] Kumar V. Singh J. Kumar P. (2019). “Heavy metals accumulation in crop plants: Sources, response mechanisms, stress tolerance and their effects,” in Contaminants in agriculture and environment: health risks and remediation. Eds. Kumar V. Kumar R. Singh J. Kumar P. (Agro Environ Media, Haridwar, India), 38–57. doi: 10.26832/AESA-2019-CAE-0161-04

[B124] Li J. Abbas M. Desoky E. S. M. Zafar S. Soaud S. A. Hussain S. S.. (2023). Analysis of metal tolerance protein (MTP) family in sunflower (*Helianthus annus* L.) and role of HaMTP10 as cadmium antiporter under moringa seed extract. Ind. Crops Prod. 202, 117023. doi: 10.1016/j.indcrop.2023.117023

[B125] Li Z. Cao Z. Ma X. Cao D. Zhao K. Zhao K.. (2024). Natural resistance-associated macrophage proteins are involved in tolerance to heavy metal Cd2^+^ toxicity and resistance to bacterial wilt of peanut (*Arachis hypogaea* L.). Plant Physiol. Biochem. 207, 108411. doi: 10.1016/j.plaphy.2024.108411 38309181

[B126] Li S. Zhou X. Li H. Liu Y. Zhu L. Guo J.. (2015). Overexpression of ZmIRT1 and ZmZIP3 enhances iron and zinc accumulation in transgenic *Arabidopsis* . PLOS One 10, e0136647. doi: 10.1371/journal.pone.0136647 26317616 PMC4552944

[B127] Lin Y. Gritsenko D. Feng S. Teh Y. C. Lu X. Xu J. (2016). Detection of heavy metal by paper-based microfluidics. Biosens. Bioelectron. 83, 256–266. doi: 10.1016/j.bios.2016.04.061 27131999

[B128] Lin Y. F. Severing E. I. Te Lintel Hekkert B. Schijlen E. Aarts M. G. (2014). A comprehensive set of transcript sequences of the heavy metal hyperaccumulator *Noccaea caerulescens* . Front. Plant Sci. 5, 261. doi: 10.3389/fpls.2014.00261 24999345 PMC4064536

[B129] Liu L. An M. M. Li X. J. Han Z. Li S. X. Li B. (2022). Molybdenum-induced effects on nitrogen absorption and utilization under different nitrogen sources in *Vitis vinifera* . J. Plant Interact. 17, 756–765. doi: 10.1080/17429145.2022.2089752

[B130] Liu H. Guo H. Jian Z. Cui H. Fang J. Zuo Z.. (2020). Copper induces oxidative stress and apoptosis in the mouse liver. Oxid. Med. Cell. Longev. 2020, 1359164. doi: 10.1155/2020/1359164 32411316 PMC7201649

[B131] Lombi E. Zhao F. J. Wieshammer G. Zhang G. McGrath S. P. (2002). *In situ* fixation of metals in soils using bauxite residue: biological effects. Environ. Pollut. 118, 445–452. doi: 10.1016/S0269-7491(01)00295-0 12009143

[B132] López-Vargas E. R. Ortega-Ortíz H. Cadenas-Pliego G. de Alba Romenus K. Cabrera de la Fuente M. Benavides-Mendoza A.. (2018). Foliar application of copper nanoparticles increases the fruit quality and the content of bioactive compounds in tomatoes. Appl. Sci. 8 (7), 1020. doi: 10.3390/app8071020

[B133] Lu S. C. (2013). Glutathione synthesis. Biochim. Biophys. Acta 1830, 3143–3153. doi: 10.1016/j.bbagen.2012.09.008 22995213 PMC3549305

[B134] Mansoor S. Ali A. Kour N. Bornhorst J. AlHarbi K. Rinklebe J.. (2023). Heavy metal induced oxidative stress mitigation and ROS scavenging in plants. Plants 12 (16), 3003. doi: 10.3390/plants12163003 37631213 PMC10459657

[B135] Mashabela M. D. Masamba P. Kappo A. P. (2023). Applications of metabolomics for the elucidation of abiotic stress tolerance in plants: a special focus on osmotic stress and heavy metal toxicity. Plants 12, 269. doi: 10.3390/plants12020269 36678982 PMC9860948

[B136] Masion A. Bertsch P. M. (1997). Aluminum speciation in the presence of wheat root cell walls: a wet chemical study. Plant Cell Environ. 20, 504–512. doi: 10.1046/j.1365-3040.1997.d01-86.x

[B137] Mattson M. P. (2008). Hormesis defined. Ageing Res. Rev. 7, 1–7. doi: 10.1016/j.arr.2007.08.007 18162444 PMC2248601

[B138] Mei H. Cheng N. H. Zhao J. Park S. Escareno R. A. Pittman J. K.. (2009). Root development under metal stress in *Arabidopsis thaliana* requires the H^+^/cation antiporter CAX4. New Phytol. 183, 95–105. doi: 10.1111/j.1469-8137.2009.02831.x 19368667

[B139] Mendes N. A. C. Cunha M. L. O. Bosse M. A. Silva V. M. Moro A. L. Agathokleous E.. (2023). Physiological and biochemical role of nickel in nodulation and biological nitrogen fixation in *Vigna unguiculata* L. Walp. Plant Physiol. Biochem. 201, 107869. doi: 10.1016/j.plaphy.2023.107869 37421847

[B140] Menguer P. K. Farthing E. Peaston K. A. Ricachenevsky F. K. Fett J. P. Williams L. E. (2013). Functional analysis of the rice vacuolar zinc transporter OsMTP1. J. Exp. Bot. 64, 2871–2883. doi: 10.1093/jxb/ert136 23761487 PMC3697945

[B141] Mishra S. Roychowdhury R. Ray S. Hada A. Kumar A. Sarker U.. (2024). Salicylic acid (SA)-mediated plant immunity against biotic stresses: an insight on molecular components and signaling mechanism. Plant Stress 11, 100427. doi: 10.1016/j.stress.2024.100427

[B142] Mishra J. Singh R. Arora N. K. (2017). Alleviation of heavy metal stress in plants and remediation of soil by rhizosphere microorganisms. Front. Microbiol. 8, 1706. doi: 10.3389/fmicb.2017.01706 28932218 PMC5592232

[B143] Mitra S. Chakraborty A. J. Tareq A. M. Emran T. B. Nainu F. Khusro A.. (2022). Impact of heavy metals on the environment and human health: Novel therapeutic insights to counter the toxicity. J. King Saud. Univ. Sci. 34, 101865. doi: 10.1016/j.jksus.2022.101865

[B144] Mousavi S. R. Shahsavari M. Rezaei M. (2011). A general overview on Manganese (Mn) importance for crops production. Aust. J. Basic Appl. Sci. 5, 1799–1803.

[B145] Muneer S. Jeong B. R. Kim T. H. Lee J. H. Soundararajan P. (2014). Transcriptional and physiological changes in relation to Fe uptake under conditions of Fe-deficiency and Cd-toxicity in roots of *Vigna radiata* L. J. Plant Res. 127, 731–742. doi: 10.1007/s10265-014-0660-0 25200143

[B146] Najeeb U. Jilani G. Ali S. Sarwar M. Xu L. Zhou W. (2011). Insights into cadmium induced physiological and ultra-structural disorders in *Juncus effusus* L. and its remediation through exogenous citric acid. J. Hazard. Mater. 186, 565–574. doi: 10.1016/j.jhazmat.2010.11.037 21159423

[B147] Nakanishi H. Ogawa I. Ishimaru Y. Mori S. Nishizawa N. (2006). Iron deficiency enhances cadmium uptake and translocation mediated by the Fe transporters OsIRT1 and OSIRT2 in rice. Soil Sci. Plant Nutr. 52, 464–469. doi: 10.1111/j.1747-0765.2006.00055.x

[B148] Niekerk L. A. Gokul A. Basson G. Badiwe M. Nkomo M. Klein A.. (2024). Heavy metal stress and mitogen activated kinase transcription factors in plants: Exploring heavy metal-ROS influences on plant signaling pathways. Plant Cell Environ. 47 (8), 2793–2810. doi: 10.1111/pce.14926 38650576

[B149] Ning X. Lin M. Huang G. Mao J. Gao Z. Wang X. (2023). Research progress on iron absorption, transport, and molecular regulation strategy in plants. Front. Plant Sci. 14, 1190768. doi: 10.3389/fpls.2023.1190768 37465388 PMC10351017

[B150] Nouet C. Motte P. Hanikenne M. (2011). Chloroplastic and mitochondrial metal homeostasis. Trends. Plant Sci. 16, 395–404. doi: 10.1016/j.tplants.2011.03.005 21489854

[B151] Oda K. Otani M. Uraguchi S. Akihiro T. Fujiwara T. (2011). Rice ABCG43 is Cd inducible and confers Cd tolerance on yeast. Biosci. Biotechnol. Biochem. 75, 1211–1213. doi: 10.1271/bbb.110193 21670506

[B152] Olaniran A. O. Balgobind A. Pillay B. (2013). Bioavailability of heavy metals in soil: impact on microbial biodegradation of organic compounds and possible improvement strategies. Int. J. Mol. Sci. 14, 10197–10228. doi: 10.3390/ijms140510197 23676353 PMC3676836

[B153] Osman H. E. Fadhlallah R. S. (2023). Impact of lead on seed germination, seedling growth, chemical composition, and forage quality of different varieties of Sorghum. J. Umm Al-Qura Univ. Appl. Sci. 9, 77–86. doi: 10.1007/s43994-022-00022-5

[B154] Pacheco D. D. R. Santana B. C. G. Pirovani C. P. de Almeida A. F. (2023). Zinc/iron-regulated transporter-like protein gene family in *Theobroma cacao* L: Characteristics, evolution, function and 3D structure analysis. Front. Plant Sci. 14, 1098401. doi: 10.3389/fpls.2023.1098401 36925749 PMC10012423

[B155] Pan D. Yi J. Li F. Li X. Liu C. Wu W.. (2020a). Dynamics of gene expression associated with arsenic uptake and transport in rice during the whole growth period. BMC Plant Biol. 20, 133. doi: 10.1186/s12870-020-02343-1 32234010 PMC7106585

[B156] Pan G. Zhao L. Li J. Huang S. Tang H. Chang L.. (2020b). Physiological responses and tolerance of flax (*Linum usitatissimum* L.) to lead stress. Acta Physiol. Plant. 42, 1–9. doi: 10.1007/s11738-020-03103-2

[B157] Pande V. Pandey S. C. Sati D. Bhatt P. Samant M. (2022). Microbial interventions in bioremediation of heavy metal contaminants in agroecosystem. Front. Microbiol. 13, 824084. doi: 10.3389/fmicb.2022.824084 35602036 PMC9120775

[B158] Pandolfini T. Gabbrielli R. Comparini C. (2006). Nickel toxicity and peroxidase activity in seedlings of *Triticum aestivum* . Plant Cell Environ. 15, 719–725. doi: 10.1111/j.1365-3040.1992.tb01014.x

[B159] Park J. Song W. Y. Ko D. Eom Y. Hansen T. H. Schiller M.. (2012). The phytochelatin transporters AtABCC1 and AtABCC2 mediate tolerance to cadmium and mercury. Plant J. 69, 278–288. doi: 10.1111/j.1365-313X.2011.04789.x 21919981

[B160] Pasricha S. Mathur V. Garg A. Lenka S. Verma K. Agarwal S. (2021). Molecular mechanisms underlying heavy metal uptake, translocation and tolerance in hyperaccumulators-an analysis: Heavy metal tolerance in hyperaccumulators. Environ. Challenges 4, 100197. doi: 10.1016/j.envc.2021.100197

[B161] Paz-Ferreiro J. Fu S. (2016). Biological indices for soil quality evaluation: perspectives and limitations. Land Degrad. Dev. 27, 14–25. doi: 10.1002/ldr.2262

[B162] Peiter E. Montanini B. Gobert A. Pedas P. Husted S. Maathuis F. J.. (2007). A secretory pathway-localized cation diffusion facilitator confers plant manganese tolerance. Proc. Natl. Acad. Sci. U.S.A. 104, 8532–8537. doi: 10.1073/pnas.0609507104 17494768 PMC1895984

[B163] Pena L. B. Azpilicueta C. E. Gallego S. M. (2011). Sunflower cotyledons cope with copper stress by inducing catalase subunits less sensitive to oxidation. J. Trace. Elem. Med. Biol. 25, 125–129. doi: 10.1016/j.jtemb.2011.05.001 21696931

[B164] Peng J. S. Gong J. M. (2014). Vacuolar sequestration capacity and long-distance metal transport in plants. Front. Plant Sci. 5, 19. doi: 10.3389/fpls.2014.00019 24550927 PMC3912839

[B165] Perea-García A. Garcia-Molina A. Andrés-Colás N. Vera-Sirera F. Pérez-Amador M. A. Puig S.. (2013). *Arabidopsis* copper transport protein COPT2 participates in the cross-talk between iron deficiency responses and low-phosphate signaling. Plant Physiol. 162, 180-194. doi: 10.1104/pp.112.212407 PMC3641201 23487432

[B166] Perincherry L. Stępień Ł. Vasudevan S. E. (2021). Cross-tolerance and autoimmunity as missing links in abiotic and biotic stress responses in plants: a perspective toward secondary metabolic engineering. Int. J. Mol. Sci. 22, 11945. doi: 10.3390/ijms222111945 34769374 PMC8584326

[B167] Phaniendra A. Jestadi D. B. Periyasamy L. (2015). Free radicals: properties, sources, targets, and their implication in various diseases. Indian J. Clin. Biochem. 30, 11–26. doi: 10.1007/s12291-014-0446-0 25646037 PMC4310837

[B168] Piñeros M. A. Magalhaes J. V. Carvalho Alves V. M. Kochian L. V. (2002). The physiology and biophysics of an aluminum tolerance mechanism based on root citrate exudation in maize. Plant Physiol. 129, 1194–1206. doi: 10.1104/pp.002295 12114573 PMC166513

[B169] Pittman J. K. Hirschi K. D. (2016). CAX-ing a wide net: Cation/H(^+^) transporters in metal remediation and abiotic stress signaling. Plant Biol. 18, 741–749. doi: 10.1111/plb.12460 27061644 PMC4982074

[B170] Podar D. Maathuis F. J. M. (2022). The role of roots and rhizosphere in providing tolerance to toxic metals and metalloids. Plant Cell Environ. 45, 719–736. doi: 10.1111/pce.14188 34622470

[B171] Pospíšil P. Yamamoto Y. (2017). Damage to photosystem II by lipid peroxidation products. Biochim. Biophys. Acta Gen. Subj. 1861, 457–466. doi: 10.1016/j.bbagen.2016.10.005 27741410

[B172] Pourrut B. Shahid M. Dumat C. Winterton P. Pinelli E. (2011). Lead uptake, toxicity, and detoxification in plants. Rev. Environ. Contam. Toxicol. 213, 113–136. doi: 10.1007/978-1-4419-9860-6_4 21541849

[B173] Priya A. K. Muruganandam M. Ali S. S. Kornaros M. (2023). Clean-up of heavy metals from contaminated soil by phytoremediation: a multidisciplinary and eco-friendly approach. Toxics 11, 422. doi: 10.3390/toxics11050422 37235237 PMC10221411

[B174] Qu T. Ma Y. Yun M. Zhao C. (2024). Transcriptome analysis revealed the possible reasons for the change of Ni resistance in *Rhus typhina* after spraying melatonin. Plants 13 (10), 1287. doi: 10.3390/plants13101287 38794358 PMC11126081

[B175] Rahoui S. Chaoui A. Ferjani E. (2010). Membrane damage and solute leakage from germinating pea seed under cadmium stress. J. Hazard. Mater. 178, 1128–1131. doi: 10.1016/j.jhazmat.2010.01.115 20185230

[B176] Rai S. Singh P. K. Mankotia S. Swain J. Satbhai S. B. (2021). Iron homeostasis in plants and its crosstalk with copper, zinc, and manganese. Plant Stress 1, 100008. doi: 10.1016/j.stress.2021.100008

[B177] Rajput V. D. Singh R. K. Verma K. K. Sharma L. Quiroz-Figueroa F. R.. (2021). Recent developments in enzymatic antioxidant defense mechanism in plants with special reference to abiotic stress. Biology 10 (4), 267. doi: 10.3390/biology10040267 33810535 PMC8066271

[B178] Rancelis V. Cesniene T. Kleizaite V. Zvingila D. Balciuniene L. (2012). Influence of cobalt uptake by *Vicia faba* seeds on chlorophyll morphosis induction, SOD polymorphism, and DNA methylation. Environ. Toxicol. 27, 32–41. doi: 10.1002/tox.20609 20549638

[B179] Rasheed A. Li H. Tahir M. M. Mahmood A. Nawaz M. Shah A. N.. (2022). The role of nanoparticles in plant biochemical, physiological, and molecular responses under drought stress: a review. Front. Plant Sci. 13, 976179. doi: 10.3389/fpls.2022.976179 36507430 PMC9730289

[B180] Rashid A. Schutte B. J. Ulery A. Deyholos M. K. Sanogo S. Lehnhoff E. A.. (2023). Heavy metal contamination in agricultural soil: environmental pollutants affecting crop health. Agronomy 13, 1521. doi: 10.3390/agronomy13061521

[B181] Raychaudhuri S. S. Pramanick P. Talukder P. Basak A. (2021). “Polyamines, metallothioneins, and phytochelatins—Natural defense of plants to mitigate heavy metals,” in Bioactive natural products. Ed. Atta-ur-Rahman F. R. S. (Elsevier, Amsterdam, Netherlands), 227–261. doi: 10.1016/B978-0-12-819487-4.00006-9

[B182] Ricachenevsky F. K. Menguer P. K. Sperotto R. A. Williams L. E. Fett J. P. (2013). Roles of plant metal tolerance proteins (MTP) in metal storage and potential use in biofortification strategies. Front. Plant Sci. 4, 144. doi: 10.3389/fpls.2013.00144 23717323 PMC3653063

[B183] Rieuwerts J. S. Thornton I. Farago M. E. Ashmore M. R. (1998). Factors influencing metal bioavailability in soils: preliminary investigations for the development of a critical loads approach for metals. Chem. Speciation Bioavailability 10, 61–75. doi: 10.3184/095422998782775835

[B184] Riyazuddin R. Nisha N. Ejaz B. Khan M. I. R. Kumar M. Ramteke P. W.. (2021). A comprehensive review on the heavy metal toxicity and sequestration in plants. Biomolecules 12, 43. doi: 10.3390/biom12010043 35053191 PMC8774178

[B185] Rodríguez Eugenio N. McLaughlin M. J. Pennock D. J. Land and Water Development Div. Food and Agriculture Organization of the United Nations (2018). Soil pollution: a hidden reality (Rome (Italy: FAO).

[B186] Ruttkay-Nedecky B. Nejdl L. Gumulec J. Zitka O. Masarik M. Eckschlager T.. (2013). The role of metallothionein in oxidative stress. Int. J. Mol. Sci. 14, 6044–6066. doi: 10.3390/ijms14036044 23502468 PMC3634463

[B187] Ryu H. W. Lee D. H. Won H. R. Kim K. H. Seong Y. J. Kwon S. H. (2015). Influence of toxicologically relevant metals on human epigenetic regulation. Toxicol. Res. 31, 1–9. doi: 10.5487/TR.2015.31.1.001 25874027 PMC4395649

[B188] Saboor A. Ali M. A. Hussain S. El Enshasy H. A. Hussain S. Ahmed N.. (2021). Zinc nutrition and arbuscular mycorrhizal symbiosis effects on maize (*Zea mays* L.) growth and productivity. Saudi J. Biol. Sci. 28, 6339–6351. doi: 10.1016/j.sjbs.2021.06.096 34759753 PMC8568715

[B189] Sabreena S. Bhat S. A. Kumar V. Ganai B. A. (2022). Phytoremediation of heavy metals: an indispensable contrivance in green remediation technology. Plants 11 (9), 1255. doi: 10.3390/plants11091255 35567256 PMC9104525

[B190] Salbitani G. Maresca V. Cianciullo P. Bossa R. Carfagna S. Basile A. (2023). Non-protein thiol compounds and antioxidant responses involved in bryophyte heavy-metal tolerance. Int. J. Mol. Sci. 24 (6), 5302. doi: 10.3390/ijms24065302 36982378 PMC10049163

[B191] Salinitro M. Mattarello G. Guardigli G. Odajiu M. Tassoni A. (2021). Induction of hormesis in plants by urban trace metal pollution. Sci. Rep. 11, 20329. doi: 10.1038/s41598-021-99657-3 34645888 PMC8514553

[B192] Sancenón V. Puig S. Mira H. Thiele D. J. Peñarrubia L. (2003). Identification of a copper transporter family in *Arabidopsis thaliana* . Plant Mol. Biol. 51, 577–587. doi: 10.1023/a:1022345507112 12650623

[B193] Sarwar N. Imran M. Shaheen M. R. Ishaque W. Kamran M. A. Matloob A.. (2017). Phytoremediation strategies for soils contaminated with heavy metals: modifications and future perspectives. Chemosphere 171, 710–721. doi: 10.1016/j.chemosphere.2016.12.116 28061428

[B194] Sasaki A. Yamaji N. Ma J. F. (2014). Overexpression of OsHMA3 enhances Cd tolerance and expression of Zn transporter genes in rice. J. Exp. Bot. 65, 6013–6021. doi: 10.1093/jxb/eru340 25151617 PMC4203134

[B195] Sattelmacher B. (2001). The apoplast and its significance for plant mineral nutrition. New Phytol. 149, 167–192. doi: 10.1046/j.1469-8137.2001.00034.x 33874640

[B196] Sawidis T. Bayçu G. Weryszko-Chmielewska E. Sulborska A. (2021). Impact of manganese on pollen germination and tube growth in lily. Acta. Agrobot. 74, 1–17. doi: 10.5586/aa.746

[B197] Schmidt S. B. Eisenhut M. Schneider A. (2020). Chloroplast transition metal regulation for efficient photosynthesis. *Trends* . Plant Sci. 25, 817–828. doi: 10.1016/j.tplants.2020.03.003 32673582

[B198] Seeda A. Abou El-Nour E.-Z. Zaghloul S. (2020). Importance of molybdenum and it diverse role in plant physiology: a review. Middle East J. Appl. Sci. 10, 228–249. doi: 10.36632/mejas/2020.10.2.23

[B199] Sethy S. K. Ghosh S. (2013). Effect of heavy metals on germination of seeds. J. Nat. Sci. Biol. Med. 4, 272–275. doi: 10.4103/0976-9668.116964 24082715 PMC3783763

[B200] Shahid M. Pourrut B. Dumat C. Nadeem M. Aslam M. Pinelli E. (2014). Heavy-metal-induced reactive oxygen species: phytotoxicity and physicochemical changes in plants. Rev. Environ. Contam. Toxicol. 232, 1–44. doi: 10.1007/978-3-319-06746-9_1 24984833

[B201] Shahzad Z. Gosti F. Frérot H. Lacombe E. Roosens N. Saumitou-Laprade P.. (2010). The five AhMTP1 zinc transporters undergo different evolutionary fates towards adaptive evolution to zinc tolerance in *Arabidopsis halleri* . PLOS Genet. 6, e1000911. doi: 10.1371/journal.pgen.1000911 20419142 PMC2855318

[B202] Sharma S. S. Dietz K.-J. Mimura T. (2016). Vacuolar compartmentalization as indispensable component of heavy metal detoxification in plants. Plant Cell Environ. 39, 1112–1126. doi: 10.1111/pce.12706 26729300

[B203] Sharma A. Sidhu G. P. S. Araniti F. Bali A. S. Shahzad B. Tripathi D. K.. (2020). The role of salicylic acid in plants exposed to heavy metals. Molecules 25, 540. doi: 10.3390/molecules25030540 31991931 PMC7037467

[B204] Shi M. Wang S. Zhang Y. Wang S. Zhao J. Feng H.. (2020). Genome-wide characterization and expression analysis of ATP-binding cassette (ABC) transporters in strawberry reveal the role of FvABCC11 in cadmium tolerance. Sci. Hortic. 271, 109464. doi: 10.1016/j.scienta.2020.109464

[B205] Shingu Y. Kudo T. Ohsato S. Kimura M. Ono Y. Yamaguchi I.. (2005). Characterization of genes encoding metal tolerance proteins isolated from *Nicotiana glauca* and *Nicotiana tabacum* . Biochem. Biophys. Res. Commun. 331, 675–680. doi: 10.1016/j.bbrc.2005.04.010 15850811

[B206] Shuting Z. Hongwei D. Qing M. Rui H. Huarong T. Lianyu Y. (2022). Identification and expression analysis of the ZRT, IRT-like protein (ZIP) gene family in *Camellia sinensis* (L.) O. Kuntze. Plant Physiol. Biochem. 172, 87–100. doi: 10.1016/j.plaphy.2022.01.008 35038675

[B207] Singh S. Parihar P. Singh R. Singh V. P. Prasad S. M. (2015). Heavy metal tolerance in plants: role of transcriptomics, proteomics, metabolomics, and ionomics. Front. Plant Sci. 6, 1143. doi: 10.3389/fpls.2015.01143 26904030 PMC4744854

[B208] Singh S. Yadav V. Arif N. Singh V. P. Dubey N. K. Ramawat N.. (2020). “Heavy metal stress and plant life: uptake mechanisms, toxicity, and alleviation,” in Plant life under changing environment. Eds. Tripathi D. K. Singh V. P. Chauhan V. ,. D. K. Sharma S. Prasad S. M. Dubey N. K. Ramawat N. (Academic Press, Cambridge, Massachusetts, USA), 271–287. doi: 10.1016/B978-0-12-818204-8.00001-1

[B209] Siripornadulsil S. Traina S. Verma D. P. Sayre R. T. (2002). Molecular mechanisms of proline-mediated tolerance to toxic heavy metals in transgenic microalgae. Plant Cell 14, 2837–2847. doi: 10.1105/tpc.004853 12417705 PMC152731

[B210] Skuza L. Szućko-Kociuba I. Filip E. Bożek I. (2022). Natural molecular mechanisms of plant hyperaccumulation and hypertolerance towards heavy metals. Int. J. Mol. Sci. 23, 9335. doi: 10.3390/ijms23169335 36012598 PMC9409101

[B211] Song W. Y. Mendoza-Cózatl D. G. Lee Y. Schroeder J. I. Ahn S. N. Lee H. S.. (2014). Phytochelatin–metal (loid) transport into vacuoles shows different substrate preferences in barley and *Arabidopsis* . Plant Cell Environ. 37, 1192–1201. doi: 10.1111/pce.12227 24313707 PMC4123957

[B212] Srivastava V. Sarkar A. Singh S. Singh P. de Araujo A. S. F. Singh R. P. (2017). Agroecological responses of heavy metal pollution with special emphasis on soil health and plant performances. Front. Environ. Sci. 5, 64. doi: 10.3389/fenvs.2017.00064

[B213] Su N. Ling F. Xing A. Zhao H. Zhu Y. Wang Y.. (2020). Lignin synthesis mediated by CCoAOMT enzymes is required for the tolerance against excess Cu in *Oryza sativa* . Environ. Exp. Bot. 175, 104059. doi: 10.1016/j.envexpbot.2020.104059

[B214] Subramanian Vignesh K. Deepe G. S. Jr. (2017). Metallothioneins: emerging modulators in immunity and infection. Int. J. Mol. Sci. 18 (10), 2197. doi: 10.3390/ijms18102197 29065550 PMC5666878

[B215] Sunda W. G. (2012). Feedback interactions between trace metal nutrients and phytoplankton in the ocean. Front. Microbiol. 3, 204. doi: 10.3389/fmicb.2012.00204 22701115 PMC3369199

[B216] Sung D. Y. Kim T. H. Komives E. A. Mendoza-Cózatl D. G. Schroeder J. I. (2009). ARS5 is a component of the 26S proteasome complex, and negatively regulates thiol biosynthesis and arsenic tolerance in *Arabidopsis* . Plant J. 59, 802–813. doi: 10.1111/j.1365-313X.2009.03914.x 19453443 PMC2830867

[B217] Sunkar R. Kaplan B. Bouché N. Arazi T. Dolev D. Talke I. N.. (2000). Expression of a truncated tobacco NtCBP4 channel in transgenic plants and disruption of the homologous *Arabidopsis* CNGC1 gene confer Pb^2+^ tolerance. Plant J. 24, 533–542. doi: 10.1046/j.1365-313x.2000.00901.x 11115134

[B218] Takahashi M. Terada Y. Nakai I. Nakanishi H. Yoshimura E. Mori S.. (2003). Role of nicotianamine in the intracellular delivery of metals and plant reproductive development. Plant Cell 15, 1263–1280. doi: 10.1105/tpc.010256 12782722 PMC156365

[B219] Tang J. Zhang J. Ren L. Zhou Y. Gao J. Luo L.. (2019). Diagnosis of soil contamination using microbiological indices: a review on heavy metal pollution. J.Environ. Manage. 242, 121–130. doi: 10.1016/j.jenvman.2019.04.061 31028952

[B220] Thirumoorthy N. Manisenthil Kumar K. T. Shyam Sundar A. Panayappan L. Chatterjee M. (2007). Metallothionein: an overview. World J. Gastroenterol. 13, 993–996. doi: 10.3748/wjg.v13.i7.993 17373731 PMC4146885

[B221] Tiwari S. Lata C. (2018). Heavy metal stress, signaling, and tolerance due to plant-associated microbes: an overview. Front. Plant Sci. 9, 452. doi: 10.3389/fpls.2018.00452 29681916 PMC5897519

[B222] Tong Y. P. Kneer R. Zhu Y. G. (2004). Vacuolar compartmentalization: a second-generation approach to engineering plants for phytoremediation. *Trends* . Plant Sci. 9, 7–9. doi: 10.1016/j.tplants.2003.11.009 14729212

[B223] Toyoda Y. Hagiya Y. Adachi T. Hoshijima K. Kuo M. T. Ishikawa T. (2008). MRP class of human ATP binding cassette (ABC) transporters: historical background and new research directions. Xenobiotica 38, 833–862. doi: 10.1080/00498250701883514 18668432

[B224] Tyagi S. Singh K. Upadhyay S. K. (2021). Molecular characterization revealed the role of catalases under abiotic and arsenic stress in bread wheat (*Triticum aestivum* L.). J. Hazard. Mater. 403, 123585. doi: 10.1016/j.jhazmat.2020.123585 32810714

[B225] Umair Hassan M. Aamer M. Umer Chattha M. Haiying T. Shahzad B. Barbanti L.. (2020). The critical role of zinc in plants facing the drought stress. Agriculture 10, 396. doi: 10.3390/agriculture10090396

[B226] Ur Rahman S. Qin A. Zain M. Mushtaq Z. Mehmood F. Riaz L.. (2024). Pb uptake, accumulation, and translocation in plants: plant physiological, biochemical, and molecular response: a review. Heliyon 10, e27724. doi: 10.1016/j.heliyon.2024.e27724 38500979 PMC10945279

[B227] van der Zaal B. J. Neuteboom L. W. Pinas J. E. Chardonnens A. N. Schat H. Verkleij J. A.. (1999). Overexpression of a novel *Arabidopsis* gene related to putative zinc-transporter genes from animals can lead to enhanced zinc resistance and accumulation. Plant Physiol. 119, 1047–1056. doi: 10.1104/pp.119.3.1047 10069843 PMC32086

[B228] Vargas-Hernandez M. Macias-Bobadilla I. Guevara-Gonzalez R. G. Romero-Gomez S. J. Rico-Garcia E. Ocampo-Velazquez R. V.. (2017). Plant hormesis management with biostimulants of biotic origin in agriculture. Front. Plant Sci. 8, 1762. doi: 10.3389/fpls.2017.01762 29081787 PMC5645530

[B229] Ventrella A. Catucci L. Piletska E. Piletsky S. Agostiano A. (2009). Interactions between heavy metals and photosynthetic materials studied by optical techniques. Bioelectrochemistry 77, 19–25. doi: 10.1016/j.bioelechem.2009.05.002 19505852

[B230] Verma R. Yadav D. Singh C. Suman A. Gaur A. (2010). Effect of heavy metals on soil respiration during decomposition of sugarcane (*Saccharum officinarum* L.) trash in different soils. Plant Soil Environ. 56, 76–81. doi: 10.17221/1773-PSE

[B231] Vidal C. Larama G. Riveros A. Meneses C. Cornejo P. (2021). Main molecular pathways associated with copper tolerance response in *Imperata cylindrica* by *de novo* transcriptome assembly. Plants 10, 357. doi: 10.3390/plants10020357 33668499 PMC7918359

[B232] Vijayaragavan M. Prabhahar C. Sureshkumar J. Natarajan A. Vijayarengan P. Sharavanan S. (2011). Toxic effect of cadmium on seed germination, growth and biochemical contents of cowpea (*Vigna unguiculata* L.) plants. Int. Multidiscip. Res. J. 1, 1–6. Available at: https://api.semanticscholar.org/CorpusID:83038868.

[B233] Wang Y. Meng Y. Mu S. Yan D. Xu X. Zhang L.. (2021b). Changes in phenotype and gene expression under lead stress revealed key genetic responses to lead tolerance in *Medicago sativa* L. Gene 791, 145714. doi: 10.1016/j.gene.2021.145714 33979680

[B234] Wang Q. Wei N. Jin X. Min X. Ma Y. Liu W. (2021a). Molecular characterization of the COPT/Ctr-type copper transporter family under heavy metal stress in alfalfa. Int. J. Biol. Macromol. 181, 644–652. doi: 10.1016/j.ijbiomac.2021.03.173 33798576

[B235] Wang Y. Wimmer U. Lichtlen P. Inderbitzin D. Stieger B. Meier P. J.. (2004). Metal-responsive transcription factor-1 (MTF-1) is essential for embryonic liver development and heavy metal detoxification in the adult liver. Faseb. J. 18, 1071–1079. doi: 10.1096/fj.03-1282com 15226267

[B236] Witkowska D. Słowik J. Chilicka K. (2021). Heavy metals and human health: possible exposure pathways and the competition for protein binding sites. Molecules 26 (19), 6060. doi: 10.3390/molecules26196060 34641604 PMC8511997

[B237] Wu X. Chen Q. Chen L. Tian F. Chen X. Han C.. (2022). A WRKY transcription factor, PyWRKY75, enhanced cadmium accumulation and tolerance in poplar. Ecotoxicol. Environ. Saf. 239, 113630. doi: 10.1016/j.ecoenv.2022.113630 35569299

[B238] Wu H. Cui H. Fu C. Li R. Qi F. Liu Z.. (2024). Unveiling the crucial role of soil microorganisms in carbon cycling: a review. Sci. Total Environ. 909, 168627. doi: 10.1016/j.scitotenv.2023.168627 37977383

[B239] Xia Y. Qi Y. Yuan Y. Wang G. Cui J. Chen Y.. (2012). Overexpression of *Elsholtzia haichowensis* metallothionein 1 (EhMT1) in tobacco plants enhances copper tolerance and accumulation in root cytoplasm and decreases hydrogen peroxide production. J. Hazard. Mater. 233, 65–71. doi: 10.1016/j.jhazmat.2012.06.047 22818176

[B240] Xiao S. Gao W. Chen Q. F. Ramalingam S. Chye M. L. (2008). Overexpression of membrane-associated acyl-CoA-binding protein ACBP1 enhances lead tolerance in *Arabidopsis* . Plant J. 54, 141–151. doi: 10.1111/j.1365-313X.2008.03402.x 18182029

[B241] Xu J. Yin H. Liu X. Li X. (2010). Salt affects plant Cd-stress responses by modulating growth and Cd accumulation. Planta 231, 449–459. doi: 10.1007/s00425-009-1070-8 19943170

[B242] Xu Y. Zhang S. Guo H. Wang S. Xu L. Li C.. (2014). OsABCB14 functions in auxin transport and iron homeostasis in rice (*Oryza sativa* L.). Plant J. 79, 106–117. doi: 10.1111/tpj.12544 24798203

[B243] Yadav S. K. (2010). Heavy metals toxicity in plants: an overview on the role of glutathione and phytochelatins in heavy metal stress tolerance of plants. South Afr. J. Bot. 76, 167–179. doi: 10.1016/j.sajb.2009.10.007

[B244] Yan A. Wang Y. Tan S. N. Mohd Yusof M. L. Ghosh S. Chen Z. (2020). Phytoremediation: a promising approach for revegetation of heavy metal-polluted land. Front. Plant Sci. 11, 359. doi: 10.3389/fpls.2020.00359 32425957 PMC7203417

[B245] Yang X. Feng Y. He Z. Stoffella P. J. (2005). Molecular mechanisms of heavy metal hyperaccumulation and phytoremediation. J. Trace Elem. Med. Biol. 18, 339–353. doi: 10.1016/j.jtemb.2005.02.007 16028496

[B246] Yang G. Fu S. Huang J. Li L. Long Y. Wei Q.. (2021a). The tonoplast-localized transporter OsABCC9 is involved in cadmium tolerance and accumulation in rice. Plant Sci. 307, 110894. doi: 10.1016/j.plantsci.2021.110894 33902855

[B247] Yang Y. R. Hou S. L. Zhang Z. W. Hu Y. Y. Ding C. Yang G. J.. (2021b). Effects of nitrogen addition on plant manganese nutrition in a temperate steppe. J. Plant Nutr. Soil. Sci. 184, 688–695. doi: 10.1002/jpln.202100144

[B248] Yao J. Sun J. Chen Y. Shi L. Yang L. Wang Y. (2020). The molecular mechanism underlying cadmium resistance in NHX1 transgenic *Lemna turonifera* was studied by comparative transcriptome analysis. Plant Cell Tiss. Organ. Cult. 143, 189–200. doi: 10.1007/s11240-020-01909-z

[B249] Yimer M. Ansari S. N. Berehe B. A. Gudimella K. K. Gedda G. Girma W. M.. (2024). Adsorptive removal of heavy metals from wastewater using cobalt-diphenylamine (Co-DPA) complex. BMC Chem. 18, 23. doi: 10.1186/s13065-024-01128-z 38287347 PMC10826029

[B250] Yuan M. Li X. Xiao J. Wang S. (2011). Molecular and functional analyses of COPT/Ctr-type copper transporter-like gene family in rice. BMC Plant Biol. 11, 69. doi: 10.1186/1471-2229-11-69 21510855 PMC3103425

[B251] Yuan L. Yang S. Liu B. Zhang M. Wu K. (2012). Molecular characterization of a rice metal tolerance protein, OsMTP1. Plant Cell Rep. 31, 67–79. doi: 10.1007/s00299-011-1140-9 21892614

[B252] Yusuf M. Fariduddin Q. Varshney P. Ahmad A. (2012). Salicylic acid minimizes nickel and/or salinity-induced toxicity in Indian mustard (*Brassica juncea*) through an improved antioxidant system. Environ. Sci. pollut. Res. 19, 8–18. doi: 10.1007/s11356-011-0531-3 21637971

[B253] Zandi P. Schnug E. (2022). Reactive oxygen species, antioxidant responses and implications from a microbial modulation perspective. Biology 11, 155. doi: 10.3390/biology11020155 35205022 PMC8869449

[B254] Zayed O. Hewedy O. A. Abdelmoteleb A. Ali M. Youssef M. S. Roumia A. F.. (2023). Nitrogen journey in plants: from uptake to metabolism, stress response, and microbe interaction. Biomolecules 13 (10), 1443. doi: 10.3390/biom13101443 37892125 PMC10605003

[B255] Zhakypbek Y. Kossalbayev B. D. Belkozhayev A. M. Murat T. Tursbekov S. Abdalimov E.. (2024). Reducing heavy metal contamination in soil and water using phytoremediation. Plants 13 (11), 1534. doi: 10.3390/plants13111534 38891342 PMC11174537

[B256] Zhan J. Zou W. Li S. Tang J. Lu X. Meng L.. (2022). OsNAC15 regulates tolerance to zinc deficiency and cadmium by binding to OsZIP7 and OsZIP10 in rice. Int. J. Mol. Sci. 23, 11771. doi: 10.3390/ijms231911771 36233067 PMC9569620

[B257] Zhang Y. Deng B. Li Z. (2018b). Inhibition of NADPH oxidase increases defense enzyme activities and improves maize seed germination under Pb stress. Ecotoxicol. Environ. Saf. 158, 187–192. doi: 10.1016/j.ecoenv.2018.04.028 29702459

[B258] Zhang J. Martinoia E. Lee Y. (2018a). Vacuolar transporters for cadmium and arsenic in plants and their applications in phytoremediation and crop development. Plant Cell Physiol. 59, 1317–1325. doi: 10.1093/pcp/pcy006 29361141

[B259] Zheng S. J. Yang J. L. He Y. F. Yu X. H. Zhang L. You J. F.. (2005). Immobilization of aluminum with phosphorus in roots is associated with high aluminum resistance in buckwheat. Plant Physiol. 138, 297–303. doi: 10.1104/pp.105.059667 15863697 PMC1104184

[B260] Zhou P. Adeel M. Shakoor N. Guo M. Hao Y. Azeem I.. (2020). Application of nanoparticles alleviates heavy metals stress and promotes plant growth: an overview. Nanomaterials 11, 26. doi: 10.3390/nano11010026 33374410 PMC7824443

[B261] Zhou B. Yao W. Wang S. Wang X. Jiang T. (2014). The metallothionein gene, TaMT3, from *Tamarix androssowii* confers Cd^2+^ tolerance in tobacco. Int. J. Mol. Sci. 15, 10398–10409. doi: 10.3390/ijms150610398 24918294 PMC4100158

[B262] Zhu C. Yu J. Cao S. Wu X. Meng W. Hou X. (2022). Transcriptomics-based analysis of genes related to lead stress and their expression in the roots of *Pogonatherum crinitum* . Front. Plant Sci. 13, 1066329. doi: 10.3389/fpls.2022.1066329 36589065 PMC9795032

